# Comparison of clustering tools in R for medium-sized 10x Genomics single-cell RNA-sequencing data

**DOI:** 10.12688/f1000research.15809.2

**Published:** 2018-12-19

**Authors:** Saskia Freytag, Luyi Tian, Ingrid Lönnstedt, Milica Ng, Melanie Bahlo

**Affiliations:** 1 Population Health and Immunity, Walter and Eliza Hall Institute of Medical Research, Parkville, Australia; 2 Department of Medical Biology, University of Melbourne, Parkville, Australia; 3 Molecular Medicine Division, Walter and Eliza Hall Institute of Medical Research, Parkville, Australia; 4 Bio21 Insititute, CSL Limited, Parkville, Australia

**Keywords:** Clustering, Single-Cell RNA-seq, Benchmarking, 10x Genomics

## Abstract

**Background:** The commercially available 10x Genomics protocol to generate droplet-based single cell RNA-seq (scRNA-seq) data is enjoying growing popularity among researchers. Fundamental to the analysis of such scRNA-seq data is the ability to cluster similar or same cells into non-overlapping groups. Many competing methods have been proposed for this task, but there is currently little guidance with regards to which method to use.

**Methods:** Here we use one gold standard 10x Genomics dataset, generated from the mixture of three cell lines, as well as multiple silver standard 10x Genomics datasets generated from peripheral blood mononuclear cells to examine not only the accuracy but also running time and robustness of a dozen methods.

**Results: **We found that Seurat outperformed other methods, although performance seems to be dependent on many factors, including the complexity of the studied system. Furthermore, we found that solutions produced by different methods have little in common with each other.

**Conclusions: **In light of this we conclude that the choice of clustering tool crucially determines interpretation of scRNA-seq data generated by 10x Genomics. Hence practitioners and consumers should remain vigilant about the outcome of 10x Genomics scRNA-seq analysis.

## Introduction

Single-cell RNA-sequencing (scRNA-seq) studies have opened the way for new data-driven definitions of cell identity and function. No longer is a cell’s type determined by arbitrary hierarchies and their respective predefined markers. Instead, a cell’s transcriptional and epigenomic profile can now be used ^1^ to accomplish this task. This is achieved using computational methods for scRNA-seq that characterize cells into novel and known cell types. Characterization consists of two steps: (i) unsupervised or semi-supervised clustering of same or similar cells into non-overlapping groups, and (ii) labeling clusters, i.e. determining the cell type, or related cell types, represented by the cluster. Here, we focus on the first step of this process.

Research into clustering has produced many algorithms for the task, including over 90 tools specifically designed for scRNA-seq ^2^. Due to the relative youth of the field, there are currently no rules guiding the application of these clustering algorithms. If tools’ performances have been tested outside synthetic scenarios, testing seems to be confined to scenarios with limited biological variability. Furthermore, most tools were developed and consequently tested only on the Fluidigm C1 protocol, despite considerable differences in throughput capabilities and sensitivities ^3^ in the different scRNA-seq platforms. Here we focus solely on clustering performance on medium-sized scRNA-seq data generated by 10x Genomics as it is currently the most widely used platform. Commercially available scRNA-seq platforms, like 10x Genomics’ Chromium, are being widely adopted due to their ease of use and relatively low cost per cell ^4^. The 10x Genomics protocol uses a droplet-based system to isolate single cells. Each droplet contains all the necessary reagents for cell lysis, barcoding, reverse transcription and molecular tagging. This is followed by pooled PCR amplification and 3’ library preparation, after which standard Illumina short-read sequencing can be applied ^5^. Unlike other commercially available scRNA-seq protocols, like Fluidigm C1, 10x Genomics allows for sequencing of thousands of cells albeit at much shallower read depths per cell, and without allowing the use of fluorescence markers to establish cell identity. As such the 10x Genomics platform is particularly suited to detailed characterization of heterogeneous tissues.

## Methods

In this study, we performed comprehensive evaluation of a dozen clustering methods ( Table 1). We focused on analysis methods available in the R language, as this is one of the most commonly used programming languages for scRNA-seq data analysis. The exception to this is the 10x Genomics software Cell Ranger. Since many methods are still being actively developed, we include assessment of program versions available in October 2017 and April 2018. Our evaluation comprised four core aspects: (i) accuracy of clustering solutions compared to a gold standard (near absolute truth, limited variability and complexity), (ii) performance of clustering methods using silver standard data (no absolute truth, realistic variability and complexity), (iii) stability of clustering solutions, and (iv) miscellaneous characteristics, such as time and practicality.

**Table 1.** Overview of the clustering tools included in this study, and several characteristics thereof.

Software	Year	Similarity Metric	Clustering Method	Ref
ascend	2017	Euclidean distance	Hierarchical clustering	7
Cell Ranger	2016	Euclidean distance	Graph-based clustering	
CIDR	2017	Imputed dissimilarity	Hierarchical clustering	8
countClust	2014	none	Grade of membership models	9
RaceID	2015	Pearson correlation	K-means clustering	10
RaceID2	2016	Pearson correlation	K-means clustering	11
RCA	2017	Pearson correlation	Supervised hierarchical clustering	12
SC3	2016	Euclidean distance	Consensus clustering	13
scran	2016	Euclidean distance	Hierarchical clustering	14
Seurat	2015	Euclidean distance	Graph-based clustering	15
SIMLR	2016	Multikernel learning	Spectral clustering	16
TSCAN	2016	none	Model-based clustering	17

### Data

***Gold standard.*** Three human lung adenocarcinoma cell lines, HCC827, H1975 and H2228, were cultured separately ^6^. The cell lines were obtained from ATCC and cultured in Roswell Park Memorial Institute 1640 medium with 10% fetal bovine serum (FBS, catalog number: 11875-176; Thermo Fisher Gibco) and 1% penicillin-streptomycin. The cells were grown independently at 37°C with 5% carbon dioxide until near 100% confluence. Before mixing cell lines, cells were dissociated into single-cell suspensions in FACS buffer (phosphate-buffered saline (PBS), catalog number: 14190-144; Thermo Fisher Gibco) with 5% FBS (catalog number: 35-076-CV; Corning), stained with propidium iodide (catalog number: P21493; Thermo Fisher FluoroPure) and 120,000 live cells were sorted for each cell line by FACS (BD FACSAria III flow cytometer, BD FACSDiva software version 7.0; BD Biology) to acquire an accurate equal mixture of live cells from the three cell lines. The resulting mixture was then processed by the Chromium Controller (10x Genomics) using single Cell 3’ Reagent Kit v2 (Chromium Single Cell 3’ Library & Gel Bead Kit v2, catalog number: 120237; Chromium Single Cell A Chip Kit, 48 runs, catalog number: 120236; 10x Genomics) (see Table 2). Afterwards the library was sequenced using Illumina NextSeq500 and V4 chemistry (NextSeq 500/550 High Output Kit v2.5, 150 Cycles, catalog number: 20024907; Iluumina) with 100bp paired end reads. RTA (version 1.18.66.3; Illumina) was used for base calling.

**Table 2.** Properties of all benchmarking datasets used in the study.

**Benchmark standard**	Gold	Silver				
Dataset		Dataset 1	Dataset 2/2a	Dataset 3/3a	Dataset 4	Dataset5
**Tissue**	Cell lines	PBMCs	PBMCs	PBMCs	PBMCs	PBMCs
**Source**	GSE111108	GSE115189	Website */ ^&^	Website ^+^/ ^−^	Website ^#^	Website ^$^
**Instrument**	Chromium	Chromium	GemCode	Chromium	GemCode	Chromium
**Number of cells**	1,039	3,372	2,691/2,700	4,337/4,340	5,419	8,381
**Total genes detected**	29,451	24,654	20,693/16,634	25,820/19,773	28,117	21,425
*After preprocessing*						
**Number of cells**	925	3,205	2,590/2,592	4,292/4,310	5,310	8,352
**Mean counts per cell**	114,426	3,818	2,605/2432	4,528/4,368	2,057	4,650
**Median genes detected** **per cell**	8,499	1,158	877/824	1,318/1,237	721	1,299

* https://support.10xgenomics.com/single-cell-gene-expression/datasets/1.0.0/pbmc3k

^&^ https://support.10xgenomics.com/single-cell-gene-expression/datasets/1.1.0/pbmc3k

^+^ https://support.10xgenomics.com/single-cell-gene-expression/datasets/1.2.0/pbmc4k

^−^ https://support.10xgenomics.com/single-cell-gene-expression/datasets/2.1.0/pbmc4k

^#^ https://support.10xgenomics.com/single-cell-gene-expression/datasets/1.1.0/pbmc6k

^$^ https://support.10xgenomics.com/single-cell-gene-expression/datasets/2.1.0/pbmc8k

***Silver standard.*** We consider five fresh human peripheral blood mononuclear cells (PBMCs) scRNA-seq datasets to be the silver standard ( Table 2). All datasets were generated using the 10x Genomics droplet system combined with Illumina sequencing. The Australian Genome Research Facility in partnership with CSL generated one dataset using the 10x Genomics Chromium system (Dataset 1). Four datasets were generated by 10x Genomics and are publicly available (Datasets 2-5). Of these, Datasets 2 and 4 were generated with an earlier version of the microfluidics instrument, the 10x Genomics GemCode Controller ( Dataset 2, Dataset 4). Datasets 3 and 5 were generated with the latest instrument, the 10x Genomics Chromium Controller ( Dataset 3, Dataset 5).

For Dataset 1, PBMCs were isolated from whole blood obtained through the Australian Red Cross Blood Service in the following manner. First, 50ml of blood was diluted using 50ml of PBS (catalog number: D8537-500ml; Sigma-Aldrich). We then added 30ml of Ficoll-Paque medium (catalog number: Catalog: 17-1440-03; GE Healthcare). We then centrifuged at room temperature for 20 minutes at 400 g and carefully removed the interface layer containing PBMCs, located between the top plasma layer and middle layer (Heraeus Multifuge 3 S-R Centrifuge, Thermo Fisher Scientific). To remove the supernatant, we further centrifuged at 400 g for 10 minutes at room temperature. This process was repeated to remove the contaminating Ficoll medium or platelets. Finally, cells were resuspended in 20ml of cell culture media with 5% FBS (RPMI-1640 Medium, catalog number: R0884-500ml, Sigma-Aldrich) and counted (Nikon Eclipse TS100 Microscope, Nikon). The resulting mixture was then processed by the Chromium Controller (10x Genomics) using single Cell 3’ Reagent Kit v2 (Chromium Single Cell 3’ Library & Gel Bead Kit v2, catalog number: 120237; Chromium Single Cell A Chip Kit, 48 runs, catalog number: 120236; 10x Genomics). Afterwards the library was sequenced using HiSeq2500 (Illumina) and V4 chemistry (HiSeq PE Cluster Kit v4 cBot, catalog number: PE-401-4001; HiSeq SBS Kit V4 50 cycles, catalog number: FC-401-4002; Illumina) with 101bp paired end reads. RTA (version 1.18.66.3, Illumina) was used for base calling.

### Preprocessing

For Datasets 1-3, we used the 10x Genomics software version 2.0.0, Cell Ranger to align to the GRCh38 (version 90) genome annotation, de-duplicate, filter barcodes and quantify genes. Note that, Cell Ranger filters any barcode that contains less than 10% of the 99 ^*th*^ percentile of total UMI counts per barcode, as these are considered to be barcodes associated with empty droplets. The barcode by design can take one of 737,000 different sequences that comprise a whitelist. This feature allows the performance of error correction when the observed barcode does not match any barcode on the whitelist due to sequencing error. Using the Bioconductor package scater ^18^ (version 1.6.3), we then removed low quality data from cells with low library size or low number of expressed gene transcripts. We also removed cells with a high mitochondrial read proportion as this can indicate apoptosis, also known as programmed cell death. Stressed cells undergoing apoptosis have an aberrant transcriptome profile in comparison to a living cell and have previously been acknowledged to adversely influence transcriptome studies ^14^.

Preprocessed versions of Datasets 2-5 were available in the R package TENxPBMCData. However, preprocessing was conducted with a CellRanger modified version of GRCh38 (version90) genome annotation resulting in slightly different versions for Dataset 2 and 3, referred to as Dataset 2a and Dataset 3a.

### Criteria for inclusion of clustering tool

We based our selection of method on the online list within www.scRNA-tools.org ^2^ in October 2017. We only considered methods with an R package that had sufficient documentation to enable easy installation and execution and had at least one preprint or publication associated with it. Note that for some of the R packages the primary focus is not clustering, but the package authors explicitly describe how their packages can be applied to achieve clustering of the scRNA-seq data. We also excluded any methods that required extensive prior information not provided in the package. We also excluded any methods that continually failed to run (e.g. Linnorm ^19^ because computation would time out and Monocle ^20^ because calculation of dispersion resulted in errors). This resulted in the evaluation of 12 methods (see Table 1 and for further details see Supplementary Table 1) in the first evaluation (R version 3.4.3). During the second evaluation of the methods (R version 3.5.0) only 11 methods were still functional. SIMLR resulted in R aborting and had to be excluded.

The aim of this study is to provide guidance for the use of clustering methods to non-experts. Hence, we used all clustering methods with their default parameters as this represents the most common use case. In the case of countClust and SIMLR parameters included the number of clusters, which we set to 3, 8 and 20 for the gold standard, silver standard datasets in evaluation 1 (R version 3.4.3) and silver standard datasets in evaluation 2 (R version 3.5.0), respectively. Marker genes were required for the analysis with scran, which we obtained by performing differential expression analyses on GSE86337 and an in-house dataset of isolated cell types in PBMCs ^21^ for the gold standard and silver standard datasets, respectively. Furthermore, we also followed upstream data handling, such as filtering of genes and normalization, as described in the documentation of the respective clustering method. We concede that it is possible that more care in the upstream data handling and selection of parameters could result in different results. However, confronted with the extremely large number of parameter choices, we believe that this evaluation suffices to identify strengths and weaknesses of each method.

### Methods for the comparison of clustering solutions

To evaluate the similarity of different clustering solutions, we rely on two different metrics. We use the adjusted Rand index (ARI) ^22^ and the normalized mutual information (NMI) ^23^, two metrics routinely applied in the field of clustering, to assess the similarity of clustering solutions or their similarity to a known truth. Both metrics can take values from 0 to 1, with 0 signifying no overlap between two groupings and 1 signifying complete overlap. These metrics are also applicable in the absence of known cluster labels. Furthermore, they share the following advantages: bounded ranges, no assumptions regarding cluster structures and symmetry.

To evaluate the performance of the different clustering methods with regards to an underlying truth, we use the ARI as well as a homogeneity score ^24^. The homogeneity score takes the value 1 when all of its clusters contain only data points that are members of a single known group. Values of this score closer to 0 indicate that clusters contain mixed known groups. Unlike ARI, this score does not penalize members of a single group being split into several clusters and thus serves as a complimentary score to the ARI. Furthermore, bounded ranges and no assumptions regarding cluster structures are properties of both the ARI with regards to ground truth and the homogeneity score.

Let *X* be a finite set of size *n*. A clustering solution *C* is a set *C*_1_, . . . , *C _k_* of non-empty disjoint subsets of *X* such that their union equals *X*. Let $C\prime =C_{1}^{\prime },...,C_{l}^{\prime }$ be a second clustering solution or the supervised labeling solution with the same properties. The contingency table *M* = ( *m _ij_* ) of the pair of sets *C*, *C*′ is a *k* × *l* matrix whose *i*, *j*-th entry equals the number of elements in the intersection of clusters *C _i_* and $C_{j}^{\prime }$:

$$m_{ij}=\left|C_{i}\cap C_{j}^{\prime }\right|,1\leq i\leq k,1\leq j\leq l.$$

***ARI***

$$R_{adj}(C,C^{\prime })=\frac{\sum_{i=1}^{k}\sum_{j=1}^{l}\left(\begin{array}{c} m_{ij}\\ 2 \end{array}\right)-t_{3}}{\left(\frac{1}{2}\left(t_{1}+t_{2}\right)-t_{3}\right)},$$

where *t*_1_ = $\sum_{i=\text{1}}^{k}\left(\begin{array}{c} \left|C_{i}\right|\\ 2 \end{array}\right)\;,t_{2}=\sum_{j=\text{1}}^{l}\left(\begin{array}{c} \left|C_{j}^{\prime }\right|\\ 2 \end{array}\right)$ and $t_{\text{3}}=\frac{2t_{1}\;t_{2}}{n\left(n-1\right)}\cdot$ For ease of notation this is referred to as ARI in the text, dropping the reference to specific pairs of sets. Furthermore, we also distinguish between ARI_truth as a comparison of a clustering solution to an underlying known or suspected truth and ARI_comp, which refers to a comparison between two clustering solutions.

***NMI***

$$NMI_{1}=\frac{I(C,C^{\prime })}{\sqrt{H(C)H(C^{\prime })}},$$

where *H*( *C* ) = *I*( *C*, *C* ) is the entropy of *C*. Note that

$$I(C,C^{\prime })=\sum_{i=1}^{k}\sum_{j=1}^{l}P(i,j)\log_{2}\frac{P(i,j)}{P(i)P(j)},$$

where $P(i,j)=\frac{m_{ij}}{n}$ and $P(i)=\frac{\left|C_{i}\right|}{n},$ is the mutual information of *C* and *C*‘.

***Homogeneity.*** Now let us assume *C*′ is the known and correct grouping of the cells. Then,

$$\text{Homogeneity}=\frac{I(C,C^{\prime })}{H(C^{\prime })}.$$

### Performance assessment

We evaluated accuracy, robustness and running time for all methods (for detailed benchmarking plan see Supplementary Table 2). For some assessments we tested methods both in R version 3.4.3 and R version 3.5.0, other assessments were only performed for one R version.

***Gold standard.*** The gold standard dataset consists of a mixture of three human lung adenocarcinoma cell lines in equal proportions. As the library preparation requires mixing these cells, the origin of each sequenced cell is technically unknown. By exploiting the genetic differences between the three different cell lines we were able to establish the cell line of origin for each cell in the gold standard dataset. To this end we first called single nucleotide variants (SNVs) in publicly available bulk RNA-seq of the same cell lines ( GSE86337) ^25^. Drawing on these SNVs, we then apply demuxlet ^26^ (version 0.0.1), which harnesses the natural genetic variation between the cell lines to determine the most likely identity of each cell. We observe almost complete concordance between the result from demuxlet and clustering of cells seen in dimension reduction visualizations of the data (compare Supplementary Figure 1). Note that the gold standard dataset was only used during the first evaluation (R version 3.4.3).

***Silver standard.*** For the silver standard data, we compared clustering solutions to a cell labeling approach by 10x Genomics ^5^ for PBMCs. This approach finds the cell type in a reference dataset which most closely resembles the expression in the cell. The reference dataset contains 11 isolated cell types sequenced using the 10x Genomics system. While this labeling does not constitute truth, it has been found to be perform well in comparison with marker-based classification ^5^. Furthermore, the proportions of cells assigned to the 11 cell types by the supervised labeling approach were consistent with the literature (see Supplementary Table 3) ^27, 28^.

Note that the first evaluation (R version 3.4.3) was performed with Datasets 1-3. The second evaluation (R version 3.5.0) was performed on Datasets 2-5, as these were available in the R package TENxPBMCData.

### Stability assessment

To test the robustness of different clustering methods we pursued a sampling strategy in terms cells. We also investigated the robustness of different methods with regards to different stringency of gene filtering. Finally, the impact of different aligners and preprocessing was assessed using all possible combinations of programs (i.e. some clustering methods did not run with scPipe output).

***Cells.*** In the first evaluation (R version 3.4.3) we used Dataset 3 for the robustness evaluation with regards to cells. We randomly sampled 3,000 cells in Dataset 3 (out of the total of 4,292 that were available after filtering), generating five (non-independent) datasets. For every combination of two datasets (10 combinations in total) we then investigated for each clustering method separately how often cells contained in all five sampled datasets were assigned to the same cluster using the ARI_comp. In the second evaluation (R version 3.5.0) we used Dataset 5. Here, we randomly sampled 4,000 cells (out of the total of 8,381 that were available after filtering), generating five (non-independent) datasets. We then repeated the evaluation procedure described above. We also investigated the variability of ARI_truth for all methods in both evaluations.

***Genes.*** Impact of gene filtering was only investigated for methods available in R version 3.5.0 during the second evaluation. We analyzed Dataset 4, as it had the most detected genes, with 10%, 20%, 30%, 40% and 50% of the most expressed genes (total counts). We investigated both the ARI_comp with regards to the clustering solution produced on a version of the dataset with no gene filtering, as well as the ARI_truth.

***Aligners and preprocessing pipelines.*** In order to assess the effect of using different preprocessing pipelines on the data, we applied the Bioconductor package scPipe ^29^ (version 1.0.6) to the raw data. Like Cell Ranger, scPipe can be used to align, de-duplicate, filter barcodes and quantify genes. Since scPipe is modular, we tried it with both the STAR ^30^ (version 020201) and Subread ^31^ (version 1.5.2) aligners. In order to ensure comparability we aligned reads to the same GRCh38 genome annotation and repeated quality control with scater. We investigated the similarity of clustering solutions applied to the differently preprocessed and aligned versions of the same dataset by ARI_comp. Note that this was only done for the evaluation with methods available in R version 3.4.3.

### Run time assessment

Each execution of a method on a dataset was performed in a separate R session. Each task was allocated as many CPU cores of a 24 core Intel(R) Xeon(R) CPU E5-2690 v3 @ 2.60GHz as specified by the default parameters, but less than 10 cores. The base::set.seed was set for all steps involving stochasticity (i.e. dimension reduction and clustering). Timings for each method include any preprocessing steps.

### Influence assessment

We also investigated what properties of each cell’s data were driving the clustering solutions produced by the different methods as well as the inferred cell labels. Properties of a cell’s data refer to features such as the number of total reads that included the cell’s barcode, the total number of detected genes found for this cell, etc. To this end, we used linear mixed models where cell data properties were predicted using the indicators for cluster membership. We predicted cell data properties and not cluster membership for modeling ease. The adjusted *R* ^2^ of these models was used to assess which properties influenced the clustering solutions. Properties investigated included: (i) the total number of detected genes, (ii) the total read count, and (iii) the percentages of reads aligning respectively to ribosomal proteins, mitochondrial genes and ribosomal RNA (only Datasets 1–3).

## Results

### Evaluation of clustering tools

**Figure 1.**
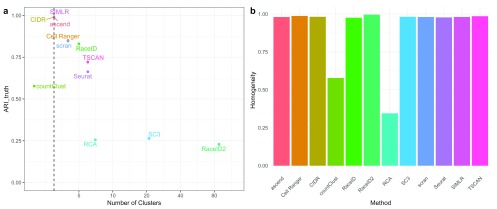
Performance on the gold standard dataset. ( **a**) ARI_truth of each method with regards to the truth versus the number of clusters. The dashed line indicates the true number of clusters. ( **b**) Homogeneity of clusters of each method, given the truth.

***Gold standard dataset.*** For the gold standard dataset consisting of three cell types, half of the tested clustering methods overestimated the true number of different cell types in the data. Methods with cluster number estimations close to the correct number of different cell types included methods with prior information, such as SIMLR, countClust and scran, as well as ascend, Cell Ranger, RaceID and CIDR ( Figure 1). The clustering solutions produced by these methods, with the exception of countClust, largely reflected cell types. This is indicated by ARI_truth >0.8. The remaining methods overestimated the number of clusters by 2 to 85 clusters, with SC3 and RaceID2 representing the extremes, both estimating more than 20 clusters (see t-SNE plots in Supplementary Figure 1 for the impact). As a consequence of the greater number of estimated clusters, the ARI_truth of the other clustering methods is lower than 0.8. To see whether these methods split cell types into several clusters or instead assign cells types randomly to clusters, we also investigate the homogeneity of the clustering solutions with respect to the known labeling. Apart from countClust and RCA, all methods have extremely high homogeneity, indicating that they split cell types into more subtypes, rather than randomly creating more cell types, which is reassuring.

**Figure 2.**
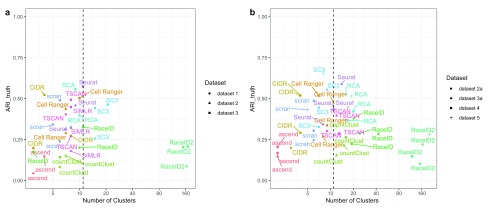
ARI_truth of each method in each dataset, as indicated by different shapes, with regards to the supervised cell labeling versus the number of clusters. The dashed line indicates the number of cell populations estimated by the supervised cell labeling approach. ( **a**) First evaluation with methods available in R 3.4.3. ( **b**) Second evaluation with methods available in R 3.5.0.

**Figure 3.**
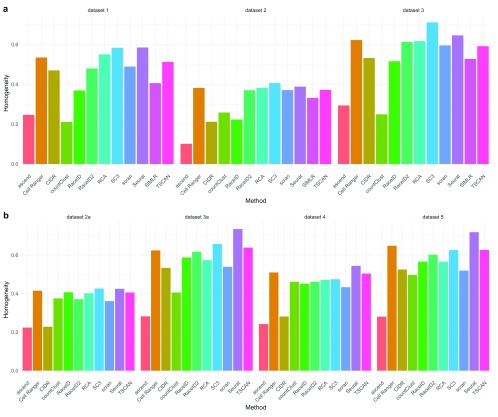
Homogeneity of clusters with regards to the inferred cell labeling for each method and each dataset. Different datasets are indicated by transparency. ( **a**) First evaluation with methods available in R 3.4.3. ( **b**) Second evaluation with methods available in R 3.5.0.

***Silver standard datasets.*** We labeled the cells in each of the silver standard datasets as one of 11 different PBMC cell populations. When using the ARI_truth to compare the likeness of the clustering solutions and the labels, no method produced solutions that were uniformly the most similar to the inferred labels ( Figure 2) in either the first or second evaluation. In both evaluations, ascend tended to estimate smaller number of clusters and consequently did not agree with the labeling. Only Seurat, SC3 and Cell Ranger achieved an ARI_truth above 0.4 for at least two datasets in each of the evaluations. All methods considerably improved their ARI_truth when we subset to more confidently labeled cells (see Supplementary Figure 2). RCA and SC3 were particularly affected, showing much greater similarity for more confidently labeled cells. We also calculated the homogeneity of each method in each dataset with respect to the inferred labeling (compare Figure 3). Generally, most methods exhibited significantly lower performance on datasets generated with the older version of the 10x Genomics technology. Most methods had much lower accuracy than for the gold standard data, indicating that most clusters represent mixtures of different inferred cell types. The exceptions are SC3’s clustering solution of Dataset 3 in the first evaluation and Seurat’s clustering solution on Datasets 3a and 5 in the second evaluation, which all achieved an homogeneity score above 0.7.

**Figure 4.**
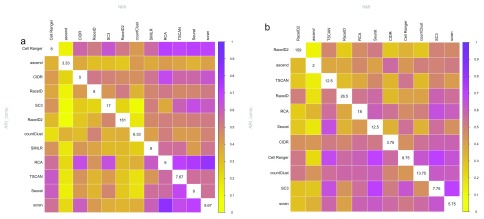
Similarity of all combinations of clustering methods as estimated by ARI_comp (lower triangle) and NMI (upper triangle) averaged over all datasets in ( **a**) evaluation 1 (R version 3.4.3) and ( **b**) evaluation 2 (R version 3.5.0). The similarity is indicated by the color; yellow indicating no similarity and purple indicating complete overlap. The diagonals give the average number of clusters estimated by each respective method. Note that methods are ordered according to similarity.

Interestingly, similar performance when compared to the labeling did not imply that cluster solutions were similar (compare Figure 4). Furthermore, similar algorithms did not result in more similar solutions. This is probably due to the vast differences in filtering and data normalization between the methods.

Most methods had comparable performance on Datasets 2/2a and 3/3a in the first and second evaluation. Consistent performance increases were only noted for countClust and Seurat (compare Supplementary Figure 3).

**Figure 5.**
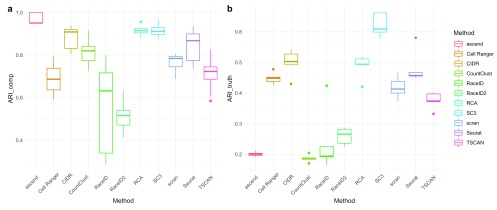
( **a**) Tukey boxplots of ARI_comp results from the comparison of clustering solutions of the same method when cell input was varied in Dataset 5. ( **b**) Tukey boxplots of ARI_truth of clustering solutions of the same method when cell input was varied in Dataset 5. Results shown are for evaluation 2 (R version 3.5.0) for results of evaluation 1 (R version 3.4.3) see Supplementary Figure 4.

**Figure 6.**
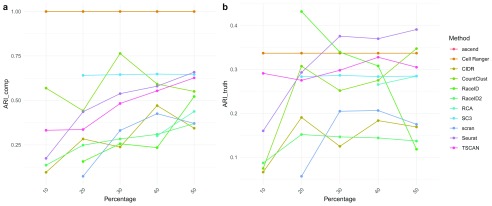
( **a**) ARI_comp of clustering solutions on Dataset 4 using 10%, 20%, 30%, 40% and 50% of the most expressed genes with respect to clustering Dataset 4 with all genes with the same method. ( **b**) ARI_truth of clustering solutions on Dataset 4 using 10%, 20%, 30%, 40% and 50% of the most expressed genes. Note that many methods could not cluster the data when few genes were available. In particular, ascend did not run.

***Stability.*** We evaluated the stability of the clustering methods by examining three different features: (i) filtering of cells Figure 5), (ii) filtering of genes ( Figure 6 and Supplementary Figure 4), and (iii) use of different aligners ( Supplementary Figure 5). When assessing the stability with regards to input in both evaluations 1 and 2, RaceID and RaceID2 did not appear very robust. Due to its reliance on reference profiles RCA is extremely robust, achieving ARI_comp above 0.9 consistently in both evaluations. In contrast, changes to gene filtering seemed to result in method specific effects, probably owing to individual filtering and normalization procedures. The performance of Seurat improved dramatically with the inclusion of more genes, whereas it deteriorated for RaceID. In contrast, both Cell Ranger and SC3 exhibited stable performance when the percentage of highly expressed genes was varied.

We also investigated how the stability of the clustering method was affected by the use of different aligners ( Supplementary Figure 5) in evaluation 1 (R version 3.4.3). In particular, we used Cell Ranger and ScPipe ^29^ with Subread ^31^, or STAR ^30^. We found that different aligners largely result in the same gene counts, but with some notable exceptions for processed pseudogenes (see Supplementary Figure 6, Supplementary Figure 7 and Supplementary Figure 8). Not all methods were able to be used in conjunction with scPipe. This included ascend and SIMLR, which failed to run, and Cell Ranger, which requires output from its own preprocessing pipeline. However we were able to evaluate eight methods. Apart from RaceID2 and RCA, all tested methods appeared robust.

**Figure 7.**
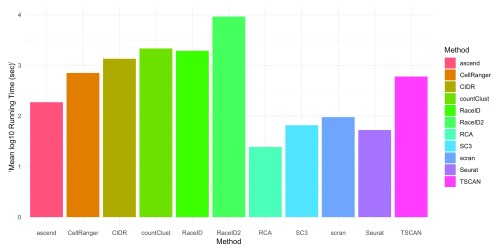
The bars indicate the average log10 run time (in seconds) of all 11 methods on Dataset 5 with 3,000 genes over 5 iterations.

***Miscellaneous properties.*** Running time varied substantially between different methods. RaceID2 took prohibitively long and thus does not lend itself to interactive analysis when applied to 10x Genomics data ( Figure 7). The fastest methods was RCA, with both taking less than 25 seconds on average for the entire dataset analysis. Considerable faster running times in evaluation 2 (R version 3.5.0) than in evaluation 1 (R version 3.4.3) were reported for Seurat and SC3 (compare Supplementary Figure 9). They were the second and third fastest methods in evaluation 2 respectively, despite offering more intermediate steps than most methods. Also note that methods differed in the quality of their documentation. For example, tools like Cell Ranger and Seurat offer detailed documentation, with many different use cases as well as tutorials (compare Supplementary Table 1). Tools, which are not found on Bioconductor, such as RaceID2, ascend and RCA have more limited documentation.

### Factors influencing clustering solutions

**Figure 8.**
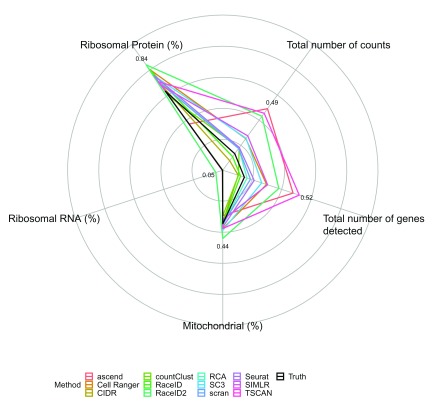
Radial plots describing the average effect of 5 cell features on the clustering solutions of different methods across the three silver standard datasets in evaluation 1 (R version 3.4.3). For every method and every feature the adjusted *R* ^2^ of the linear model fitting the feature by the clustering solution is presented.

The variation in the percentage of reads aligning to ribosomal protein genes strongly predicted all clustering solutions as well as the inferred cell labels (see Figure 8, Supplementary Figure 10, Supplementary Figure 11). Expression of ribosomal protein genes has been successfully used to discriminate cell types belonging to different hematopoietic lineages ^32^. Hence, it may be the case that overall mRNA amount of ribosomal protein genes can also serve as a discriminator. Furthermore, differences in abundance of ribosomal protein genes are likely to drive variation in PBMC scRNA-seq datasets, as they typically account for a large proportion of reads (around 40% in all three datasets). In combination with ribosomal protein genes being less affected by dropout due to their relatively high expression, it is perhaps unsurprising that clustering solutions of all methods foremost reflect differences in the amount of ribosomal protein genes between cells.

Most methods’ solutions were much more driven by the total number of detected genes and total number of counts than the inferred solution. TSCAN was particularly affected ( *R* ^2^ = 0.52 in evaluation 1 and *R* ^2^ = 0.68 in evaluation 2), but for RaceID2 similar effects were observed. It can be speculated that this strong influence of total number of features and total number of counts on their clustering solutions points to a failure to appropriately normalize the data.

## Discussion

**Figure 9.**
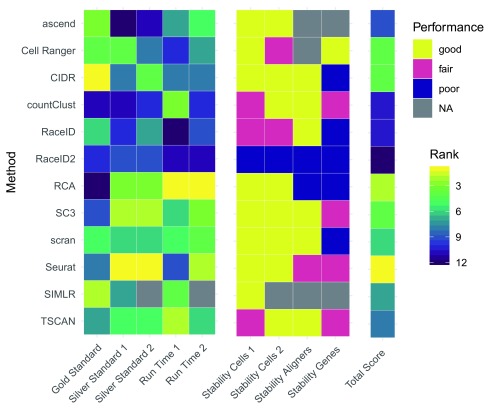
Summary of the performance of each method across all evaluations. Note that 1 refers to evaluation 1 (R version 3.4.3) and 2 refers to evaluation 2 (R version 3.5.0).

We also summarized the performance of each method across all evaluations (see Figure 9). This summary suggests that Seurat provides the best clustering solutions for 10x Genomics scRNA-seq data in terms of running time, robustness and accuracy. The next best performing methods were RCA, SC3, Cell Ranger and CIDR. However, it should be noted that RCA performed particularly poorly on the gold standard dataset. This highlights that RCA’s performance hinges on the studied cell types being represented in the reference used during the supervised clustering approach. These results closely mimic benchmarking results observed by Duò *et al.* ^33^ on independent silver standard and simulated datasets across multiple single cell technologies.

We also investigated whether properties of the clustering method correlated with their performance. We found that neither the type of clustering method used nor the similarity metric used seemed to correlate with the performance. However, our ability to identify patterns might have been impacted by the small sample size. A recent paper by Kim *et al.* ^34^, which systematically studied the effect of different similarity metrics on performance of scRNA-seq clustering methods, found that correlation-based similarity metrics outperformed distance-based metrics.

## Conclusion

Most biological conclusions obtained from droplet-based scRNA-seq data crucially rely on accurate clustering of cells into homogeneous groups. Indeed, one can argue that it is the very act of clustering that unlocks the technology’s potential for discovery. Therefore it is not surprising that according to several repositories, such as www.omicstools.org and www.scRNA-tools.org ^2^, many of the tools developed for scRNA-seq specifically focus on clustering. With so many choices, it is thus important to evaluate their performance for droplet based protocols, such as 10x Genomics, specifically.

In this study, we presented our evaluation of a dozen clustering method on scRNA-seq 10x Genomics data. The results of our investigations will be useful for method users, as we provide practical guidelines. Nonetheless, our evaluation has several limitations:

• Inclusion of methods limited to R packages and methods published before October 2017
• Parameter selection limited to defaults
• No assessment of robustness to noise and parameter changes
• No assessment of ability to discover rare cell populations
• Evaluation of more silver standard datasets from systems other than PBMCs
• No evaluation of ability to deal with batch effects or other more complex designs
• No evaluation of quality of code and documentation
• No assessment of scalability of methods

While Seurat performed slightly better than the next best methods, in our opinion, the choice of clustering method should be informed by the user’s familiarity with statistical concepts and R programming. Many methods, including Seurat, require the user to make informed parameter choices and occasionally troubleshoot code. Methods requiring no parameter choices, like Cell Ranger, may offer a better choice for non-experts.

In general, we recommend that practitioners and consumers of results generated from 10x Genomics scRNA-seq data alike remain vigilant about the outcome of their analysis, and acknowledge the variability and likelihood of undesired influences. The choice of clustering tool for scRNA-seq data generated by the 10x Genomics platform crucially determines interpretation. Hence, we suggest using several clustering methods ideally with multiple parameter choices on 10x Genomics scRNA-seq data in order to ensure that biological results are not artifacts of method or parameter choice. This should help guard against subjective interpretation of the data and thus increase robustness of and confidence in results.

## Data availability

Repository: Gold Standard Dataset. Single cell profiling of 3 Human Lung Adenocarcinoma cell lines, GSE111108 Repository: Silver Standard Dataset 1. Single cell profiling of peripheral blood mononuclear cells from healthy human donor, GSE115189

Repository: Silver Standard Dataset 2. 3k PBMCs from a Healthy Donor, Version 1.0.0: https://support.10xgenomics.com/single-cell-gene-expression/datasets/1.0.0/pbmc3k, Version 1.1.0: https://support.10xgenomics.com/single-cell-gene-expression/datasets/1.1.0/pbmc3k

Repository: Silver Standard Dataset 3. 4k PBMCs from a Healthy Donor, Version 1.2.0 https://support.10xgenomics.com/single-cell-gene-expression/datasets/1.2.0/pbmc4k, Version 2.1.0 https://support.10xgenomics.com/single-cell-gene-expression/datasets/2.1.0/pbmc4k

Repository: Silver Standard Dataset 4. 6k PBMCs from a Healthy Donor, https://support.10xgenomics.com/single-cell-gene-expression/datasets/1.1.0/pbmc6k

Repository: Silver Standard Dataset 5. 8k PBMCs from a Healthy Donor, https://support.10xgenomics.com/single-cell-gene-expression/datasets/2.1.0/pbmc8k

We also provide versions in the R Single-CellExperiment format of all datasets at https://github.com/bahlolab/cluster_benchmark_data

## Software availability

**All code is available for download at:** https://github.com/SaskiaFreytag/cluster_benchmarking_code.

**Archived code at time of publication:** 10.5281/zenodo.2008645

**License:** MIT License

## Consent

Written informed consent for publication of the participant’s transcriptomic information was obtained (Australian Red Cross Blood Service Supply Agreement 1803VIC-07).
