# Supplementary material for: Comparison of clustering tools in R for medium-sized 10x Genomics single-cell RNA-sequencing data
Source: F1000Res. 2018 Dec 19;7:1297. Originally published 2018 Aug 15. [Version 2] doi: 10.12688/f1000research.15809.2 (PMC6124389; doi:10.12688/f1000research.15809.2)
Supplement: Supplementary file 1 [file f1000research-7-19170-s0000.tgz › ab8d23e4-f9e2-41e4-a057-43630939796b_Supp_Figures.pdf]

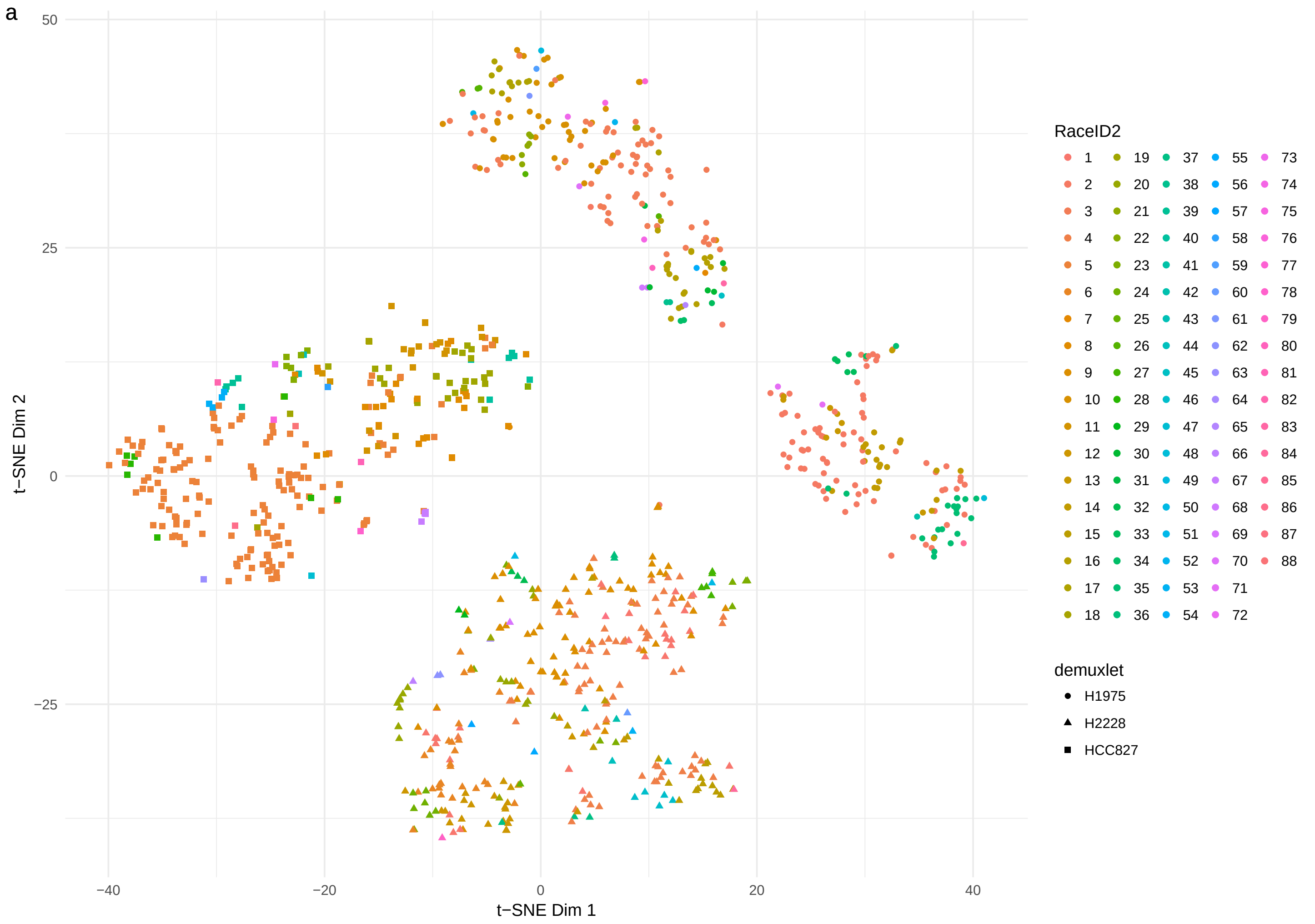
a
t−SNE Dim 1
t−SNE Dim 2
RaceID2
demuxlet
H1975
H2228
HCC827

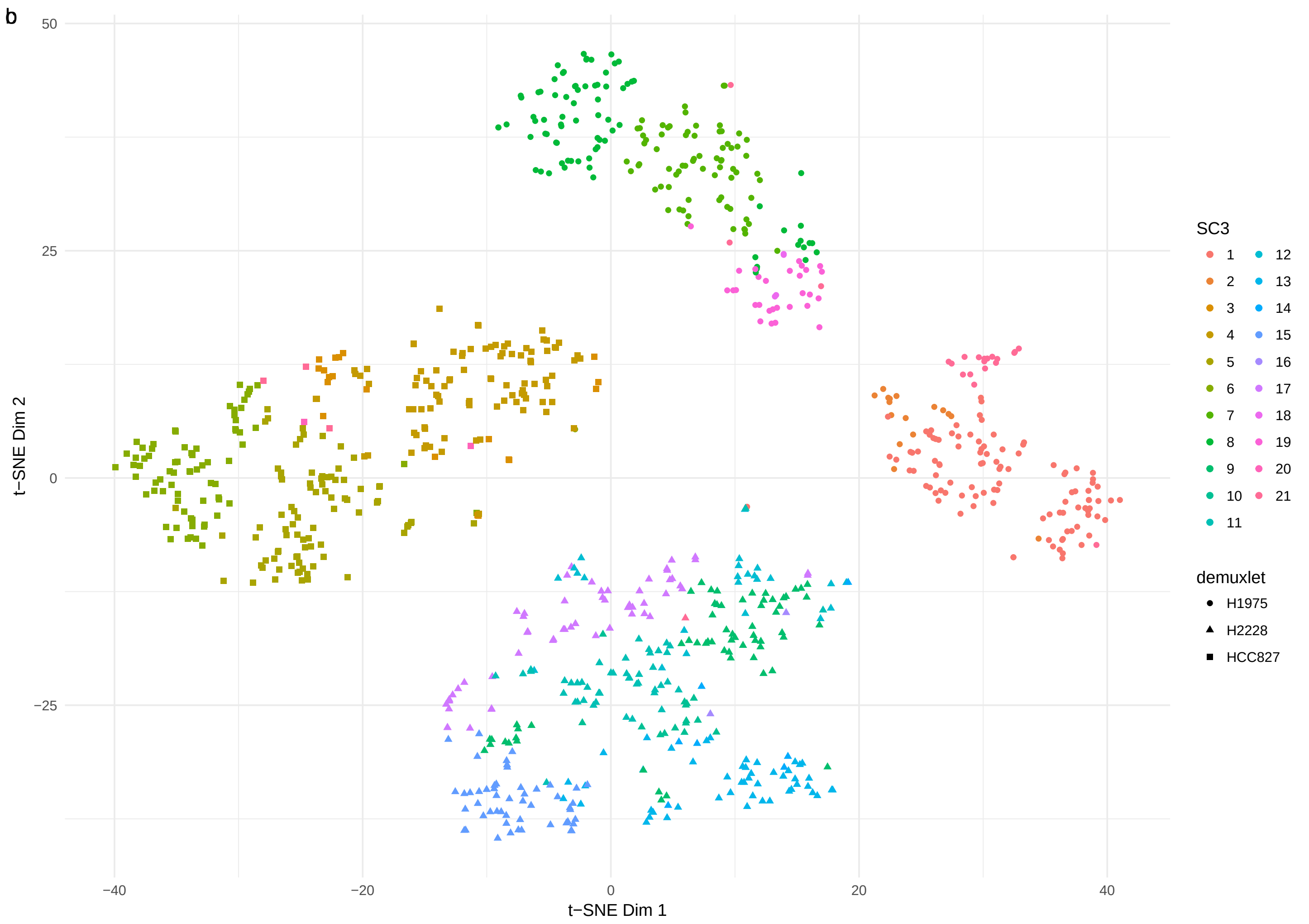
b
50
25
0
−25
t−SNE Dim 2
−40
−20
0
20
40
t−SNE Dim 1
SC3
1
2
3
4
5
6
7
8
9
10
11
12
13
14
15
16
17
18
19
20
21
demuxlet
H1975
H2228
HCC827

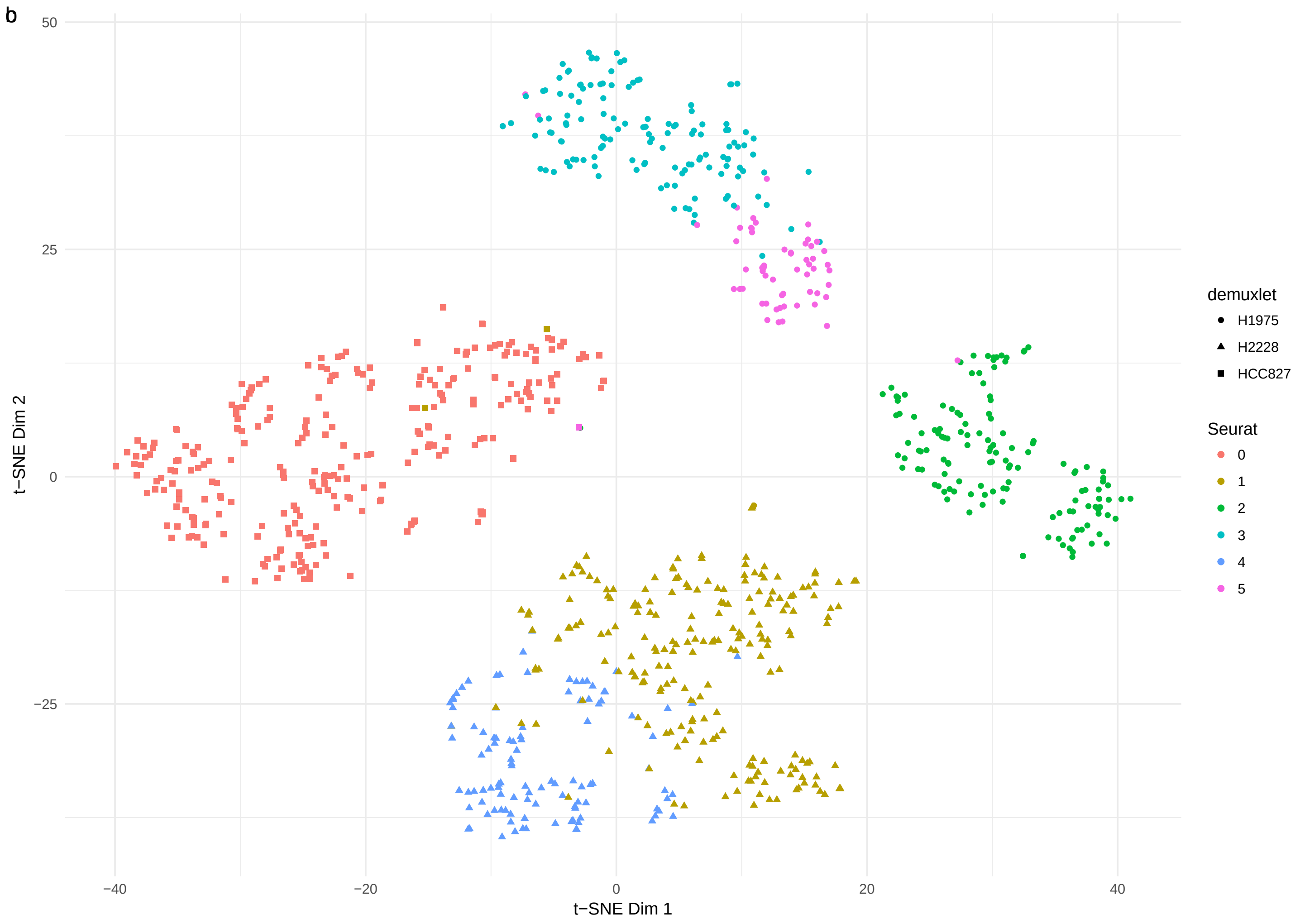

Supplementary Figure 1: T-SNE plot of the gold standard data after filtering and normalization with the package scater. Shapes indicate the cell identity as established by demuxlet. The colors indicate in a) RaceID2 clustering, b) SC3 clustering and in c) Seurat clustering. It can be observed that these three programs present different degrees of complexity.

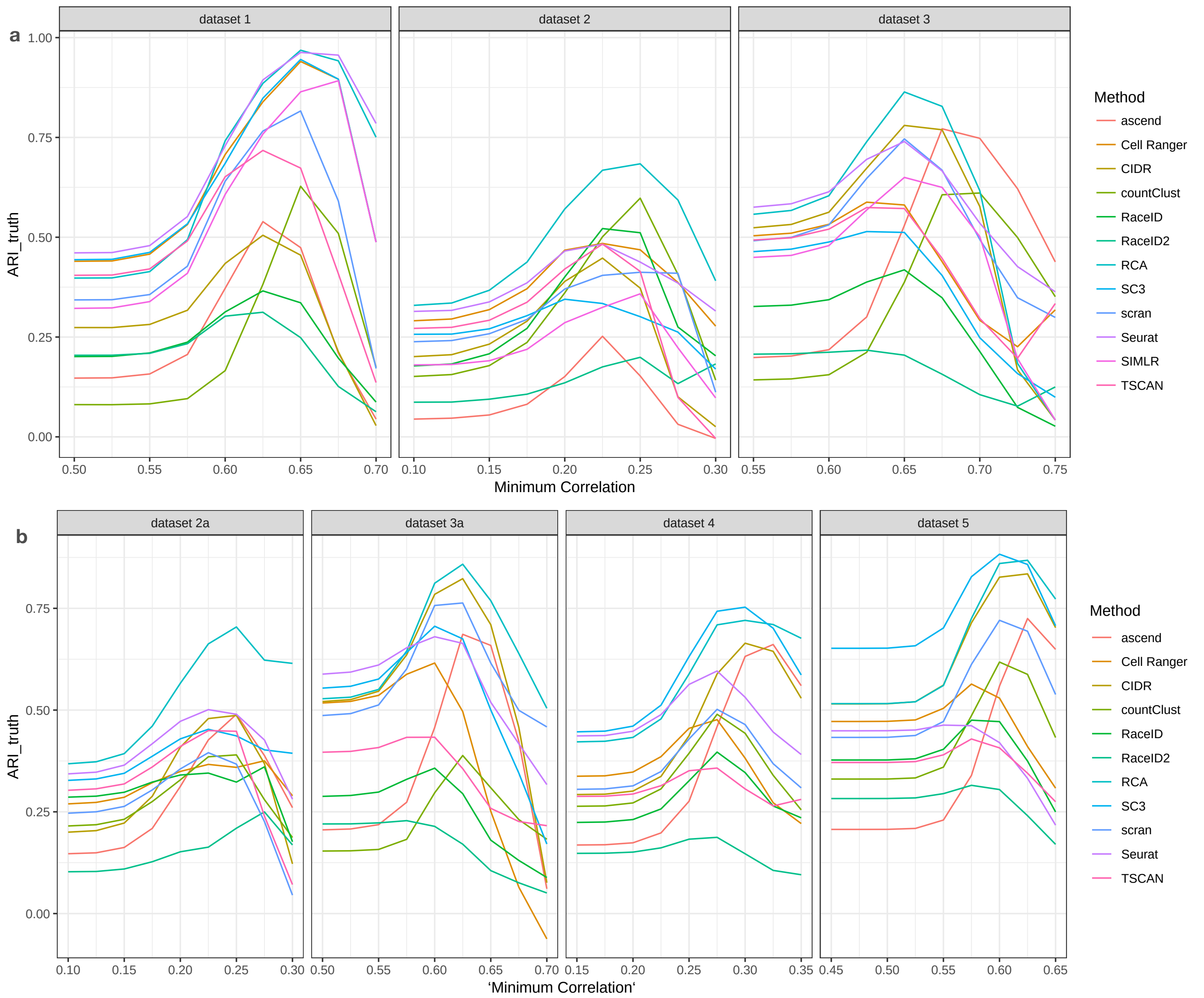

Supplementary Figure 2: ARI_truth of each methods on all silver standard datasets given the subset of the cells that reached respective minimum correlation using the cell labeling approach by Zheng et al. (a) Evaluation 1 (R version 3.4.3) (b) Evaluation 2 (R version 3.5.0)

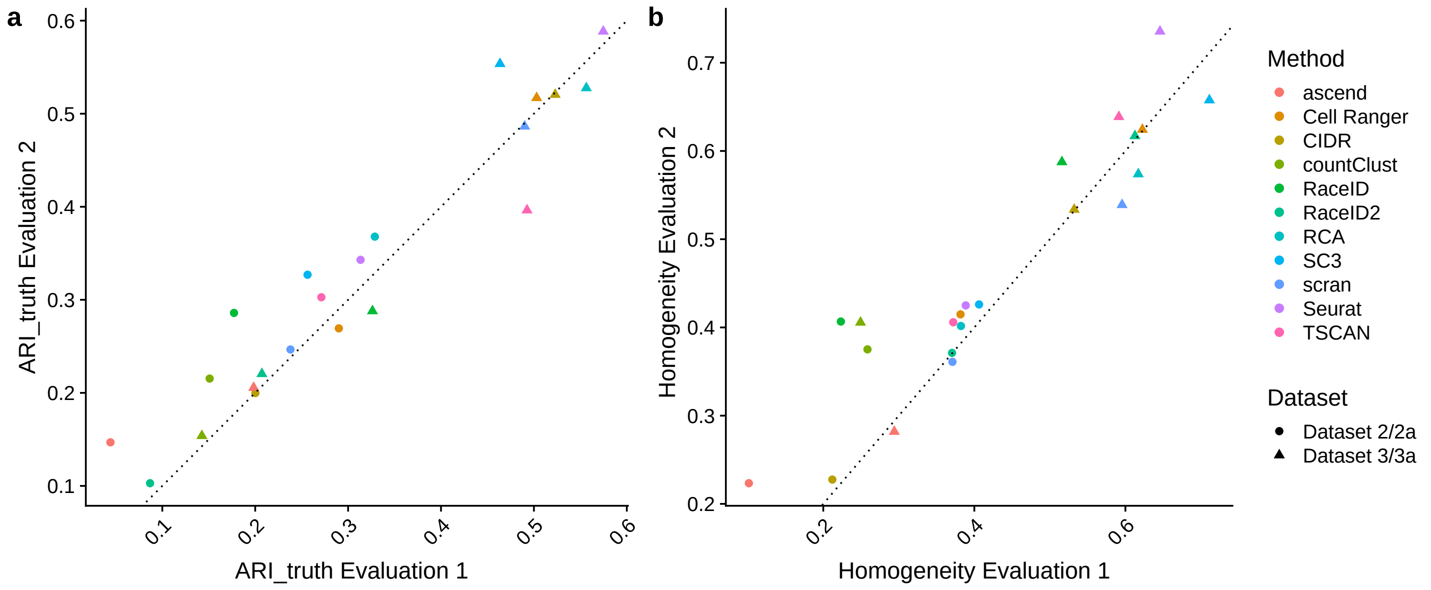

Supplementary Figure 3: Comparison of evaluation 1 and evaluation 2 in Dataset 2/2a and Dataset 3/3a. (a) ARI_truth in evaluation 1 (R version R 3.4.3) versus ARI_truth in evaluation 2 (R version 3.5.0) of each methods for Datasets 2/2a and 3/3a. (b) Homogeneity in evaluation 1 (R version R 3.4.3) homogeneity in evaluation 2 of each methods for Datasets 2/2a and 3/3a (R version 3.5.0). The diagonal, where methods performed equally well in evaluation 1 and 2, is indicated by the dotted line.

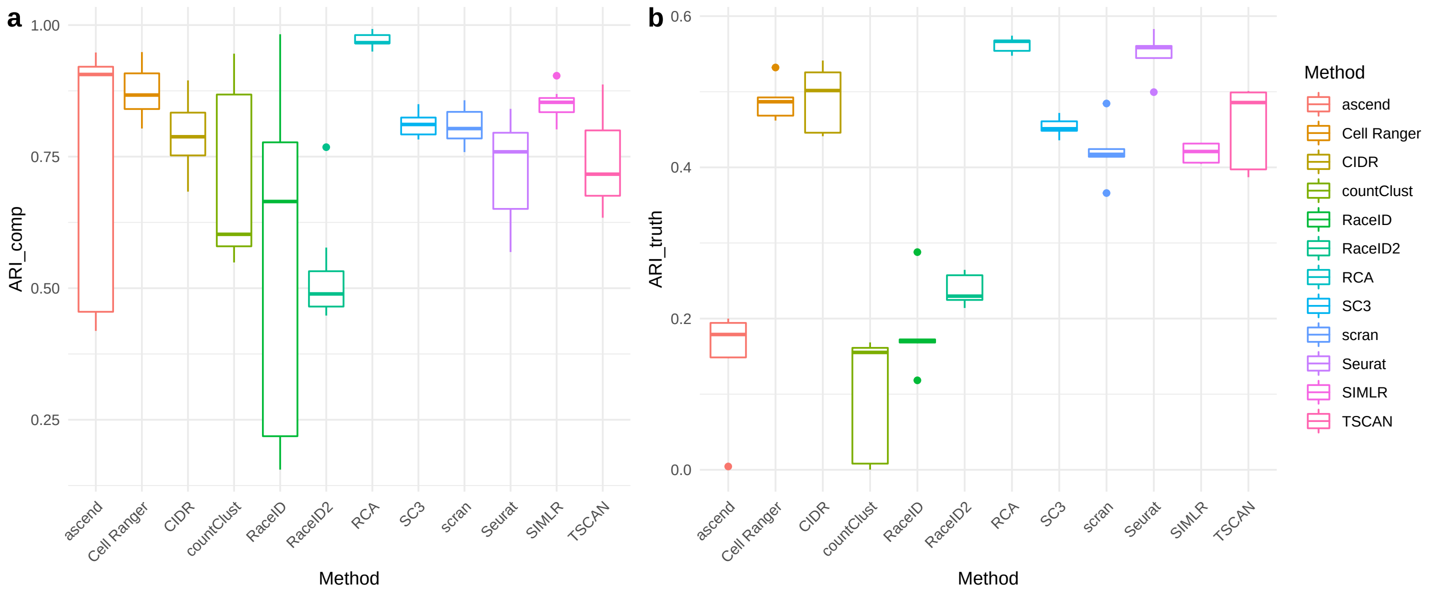

Supplementary Figure 4: Robustness with regards to changes in cell input. (a) Tukey boxplots of ARI_comp results from the comparison of clustering solutions in evaluation 1 of the same method when cell input was varied in Dataset 3. (b) Tukey boxplots of ARI_truth of clustering solutions in evaluation 1 when cell input was varied in Dataset 3.

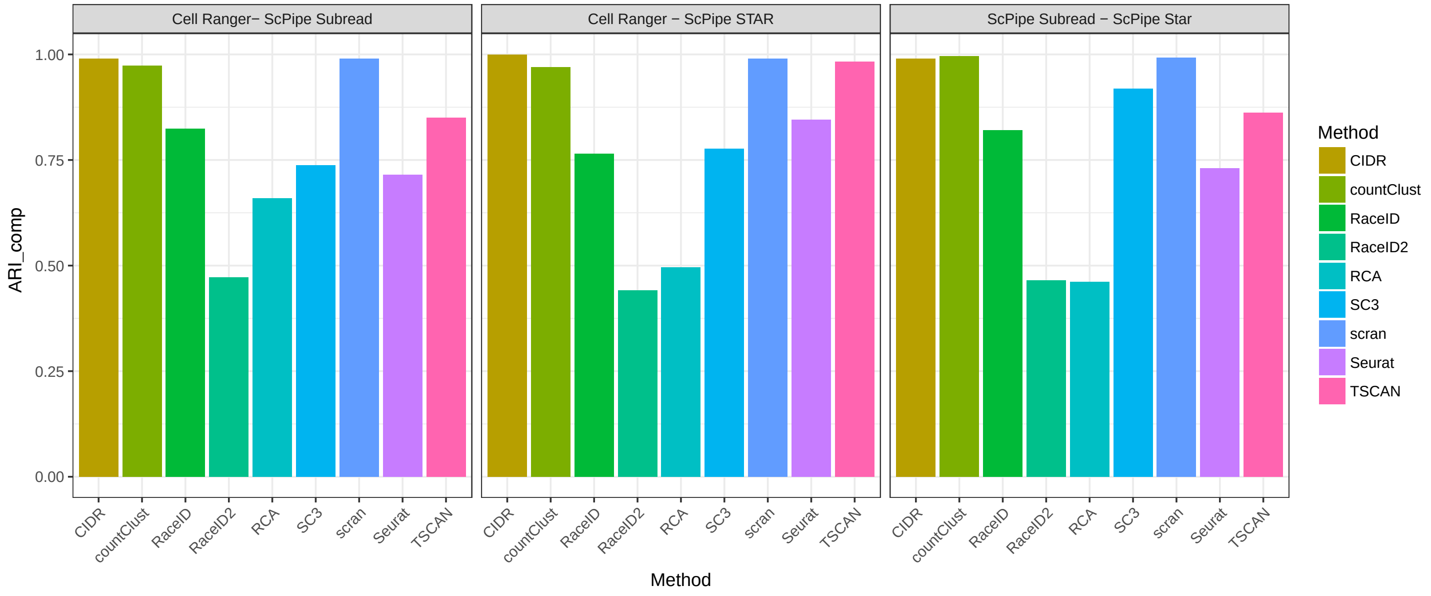

Supplementary Figure 5: ARI_comp results from the comparison of clustering solutions of the same method used on datasets processed with different aligners. Only the subset of nine methods that worked in conjunction with all three aligners are shown.

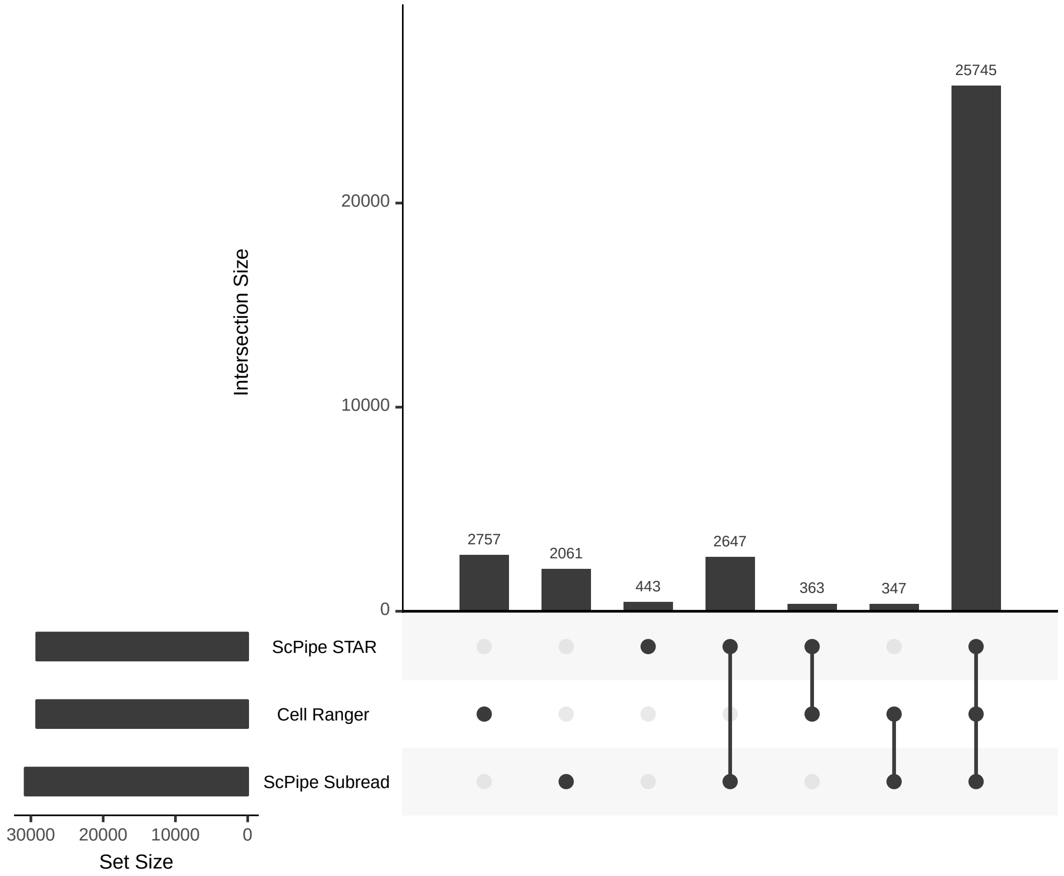

Supplementary Figure 6: UpSeTR Venn diagram to compare of number genes detected in the gold standard dataset by different programs used for preprocessing. The vast majority of genes are detected by all programs.

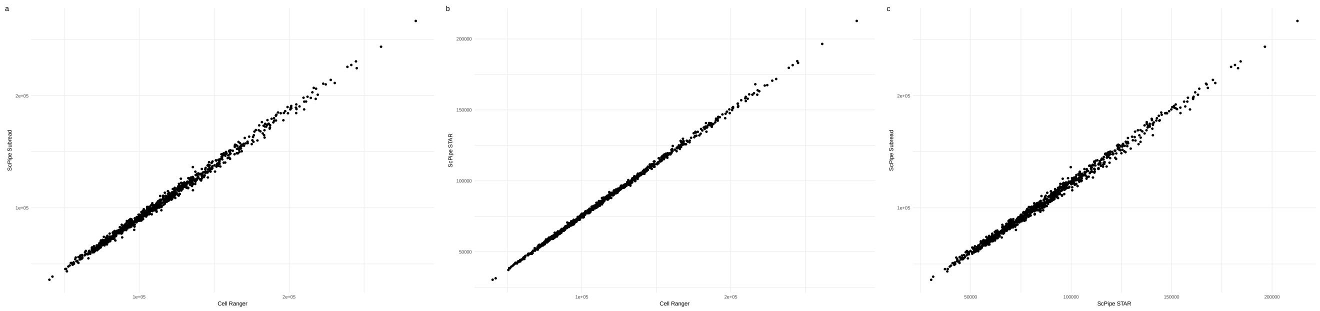

Supplementary Figure 7: Total counts for the same barcode as measured when processed with a) Cell Ranger versus ScPipe Subread b) Cell Ranger versus ScPipe STAR and c) ScPipe STAR versus ScPipe STAR. Only barcodes are shown that appear in all three versions of the processed dataset.

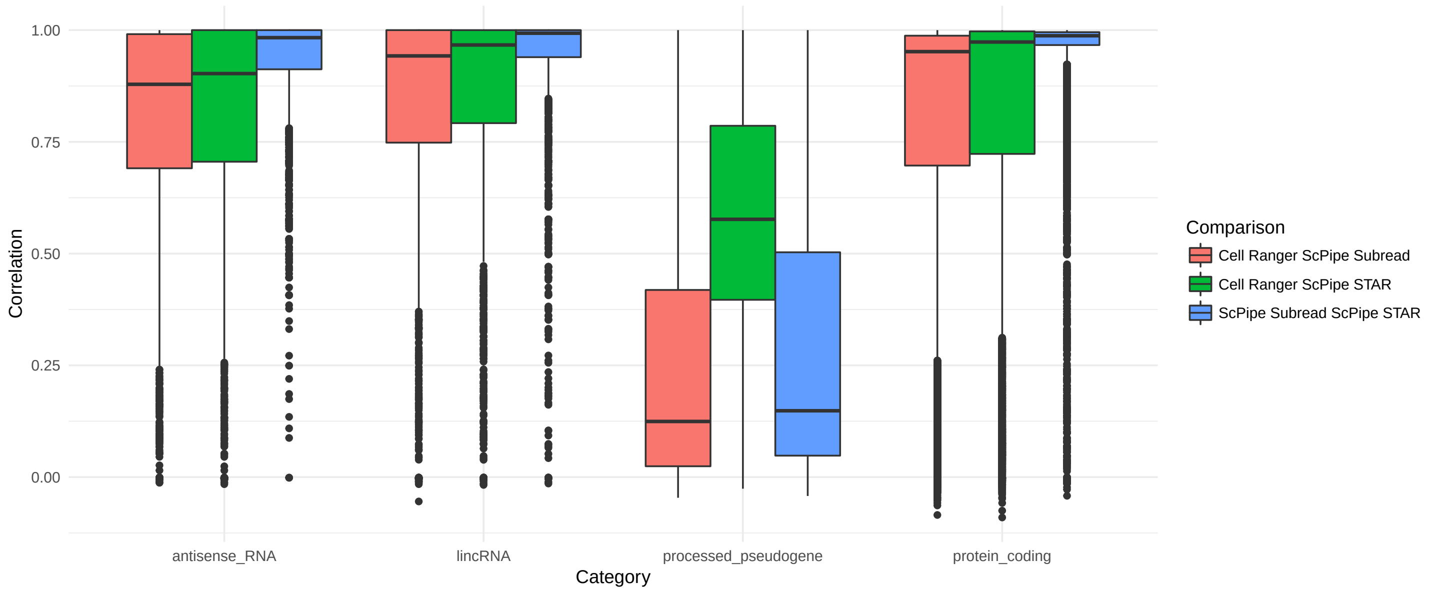

Supplementary Figure 8: Boxplots showing the correlations between gene counts of particular gene category for all three comparisons between preprocessing programs used on gold standard dataset. Processed pseudogenes' counts seem to differ depending on program used.

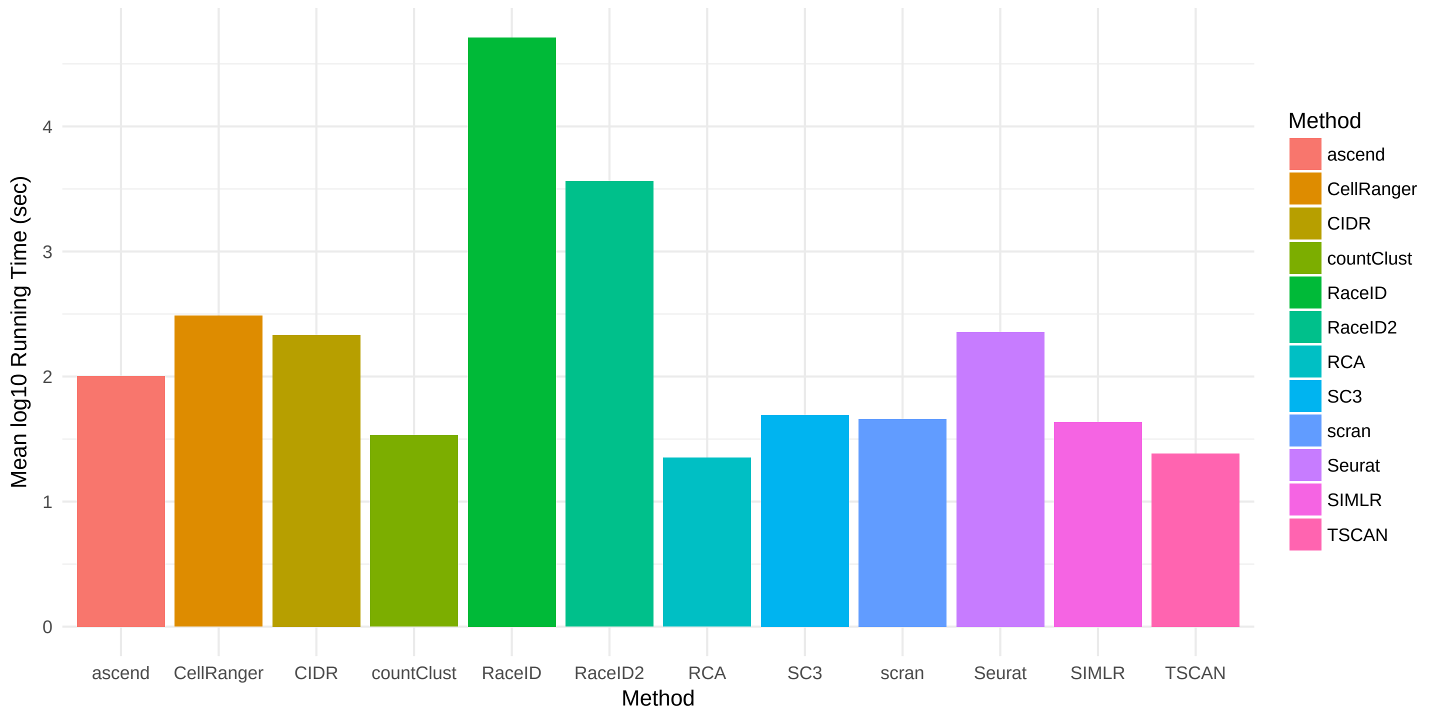

Supplementary Figure 9: The bars indicate the average log10 run time (in seconds) of all 12 methods on Dataset 1 with 29,151 genes over 10 iterations.

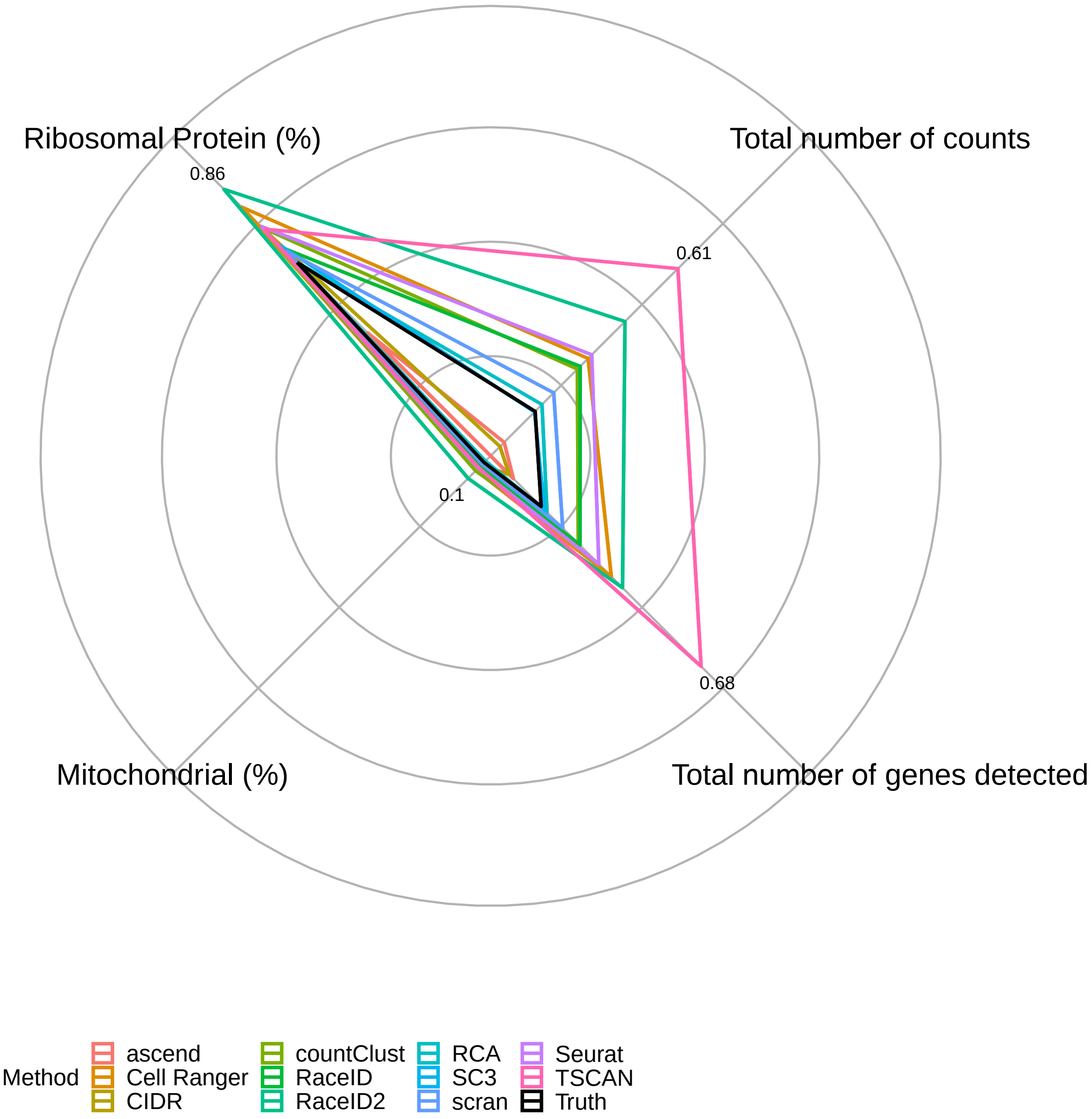

Supplementary Figure 10: Radial plots describing the average effect of 4 cell features on the clustering solutions of different methods across the four silver standard datasets in evaluation 2 (R version 3.5.0). For every method and every feature the adjusted R^2 of the linear model fitting the feature by the clustering solution is presented.

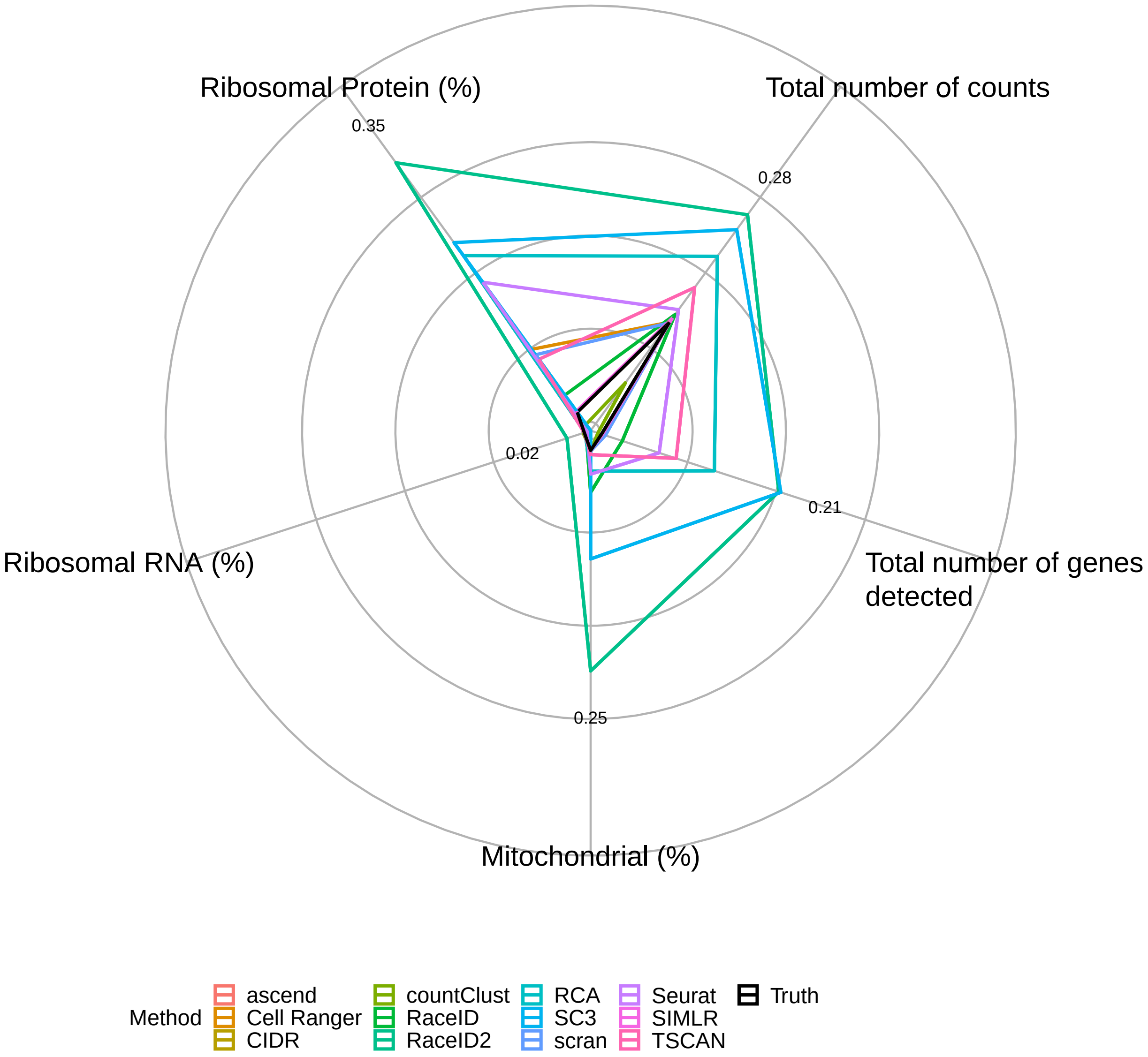

Supplementary Figure 11: Radial plots describing the average effect of 5 cell features on the clustering solutions of different methods on the gold standard datasets. For every method and every feature the adjusted R^2 of the linear model fitting the feature by the clustering solution is presented.
