# Supplementary material for: Comparison of clustering tools in R for medium-sized 10x Genomics single-cell RNA-sequencing data
Source: F1000Res. 2018 Dec 19;7:1297. Originally published 2018 Aug 15. [Version 2] doi: 10.12688/f1000research.15809.2 (PMC6124389; doi:10.12688/f1000research.15809.2)
Supplement: Supplementary file 2 [file f1000research-7-19170-s0001.tgz › 34ca038d-e372-4e49-835f-90acce20d669_Supp_Tables.pdf]

Supplementary Table 1: Overview of the clustering tools included in this study, and further characteristics thereof.

**Software**	**R 3.4.3**	**R 3.5.0**	**Description**	**Properties**
`ascend`	version 0.4.0	version 0.9.0	Adjusted RLE normalization followed by hierarchical clustering and merging	Gene filtering, limited documentation, medium user interaction
`Cell Ranger`	version 2.0.0	version 2.2.0	Graph-based clustering on the first 10 principal components	Gene filtering, detailed documentation, no user interaction
`CIDR`	version 0.1.5	version 0.1.5	Imputation of potential dropout genes followed by hierarchical clustering on first 4 principal components	Gene filtering, imputation good documentation, high user interaction
`countClust`	version 1.4.1	version 1.9.1	Likelihood models to estimate a specified number of multinomial distributions	Requires number of clusters, limited documentation, medium user interaction
`RaceID`		version 0.1.1	Two iterations of k-means clustering with merging of outlier cells and identification of rare cell types in last step	Gene filtering, limited documentation, little user interaction
`RaceID2`			More advanced version of RaceID based on UMIs	Gene filtering, limited documentation, little interaction
`RCA`	version 1.0	version 1.0	Rudimentary filtering then projection onto reference datasets consisting of profiles of isolated cell types	Gene filtering, reference dataset required, limited documentation, medium user interaction
`SC3`	version 1.7.7	version 1.9.1	Rudimentary filtering followed by ensembl clustering method	Gene filtering, good documentation,medium user interaction
`scran`	version 1.6.9	version 1.9.33	Library scale normalization by cell pools followed by hierarchical clustering on rank correlation-based distances of marker genes	Gene filtering, requires marker genes, detailed documentation, high user interaction
`Seurat`	version 2.3.0	version 2.3.4	Normalization using mitochondrial RNA followed by PCA of highly variable genes and then graph-based clustering	Gene filtering, detailed documentation, high user interaction
`SIMLR`	version 1.4.1	version 1.7.2	Multikernel learning finds best fit and forces blocks in similarity matrix to address dropouts then applies spectral clustering	Requires number of clusters, good documentation, little user interaction
`TSCAN`	version 1.16.0	version 1.19.0	In-silico pseudo time reconstruction with a cluster-based minimum spanning tree approach to order cells	Good documentation, little user interaction

Supplementary Table 2: Assessment of clustering methods by type and datasets used.

		Performance	Stability	Run Time	Influence
Gold Standard		R 3.4.3	Aligners (not run: `ascend`, `SIMLR`, `Cell Ranger`)		✓
Silver Standard	Dataset 1	R.3.4.3		✓	✓
	Dataset 2	R.3.4.3			✓
		R 3.5.0 (not run: `SIMLR`)			✓
	Dataset 3	R 3.4.3	Cells		✓
		R 3.5.0 (not run: `SIMLR`)			✓
	Dataset 4	R 3.5.0 (not run: `SIMLR`)	Genes (not run: `ascend`, `SIMLR`)		✓
	Dataset 5	R 3.5.0 (not run: `SIMLR`)	Cells (not run: `SIMLR`)	✓	✓

Supplementary Table 3: Proportion of cell types in different silver standard datasets as estimated by supervised cell labeling.

Cell type	Dataset 1	Dataset2a	Dataset 2	Dataset 3	Dataset3a	Dataset 4	Dataset5
CD14+ Monocyte	0.14	0.22	0.22	0.25	0.26	0.23	0.26
CD19+ B	0.13	0.11	0.10	0.14	0.14	0.13	0.14
CD34+	0.01	0.04	0.05	0.00	0.00	0.01	0.00
CD4+/CD25 T Reg	0.06	0.14	0.12	0.08	0.07	0.13	0.10
CD4+/CD45RA+/CD25- Naive T	0.08	0.06	0.08	0.09	0.10	0.07	0.09
CD4+/CD45RO+ Memory	0.15	0.13	0.13	0.08	0.09	0.09	0.07
CD4+ T Helper2	0.04	0.01	0.02	0.04	0.01	0.02	0.01
CD56+ NK	0.13	0.11	0.11	0.05	0.06	0.10	0.05
CD8+/CD45RA+ Naive Cytotoxic	0.16	0.04	0.04	0.15	0.14	0.06	0.15
CD8+ Cytotoxic T	0.12	0.08	0.07	0.11	0.11	0.12	0.11
Dendritic	0.01	0.04	0.05	0.03	0.03	0.04	0.03
