# Supplementary material for: Comparison of clustering tools in R for medium-sized 10x Genomics single-cell RNA-sequencing data
Source: F1000Res. 2018 Dec 19;7:1297. Originally published 2018 Aug 15. [Version 2] doi: 10.12688/f1000research.15809.2 (PMC6124389; doi:10.12688/f1000research.15809.2)
Supplement: Peer review history

# Peer review: Referee response for version 2

**DOI:** 10.5256/f1000research.19170.r42132

**Published:** 2019-01-11

**Authors:** Shila Ghazanfar

**Affiliations:** 1 School of Mathematics and Statistics, University of Sydney, Sydney, NSW, Australia

The authors have provided excellent responses to reviewer comments, leading to a more comprehensive and useful manuscript.

I have read this submission. I believe that I have an appropriate level of expertise to confirm that it is of an acceptable scientific standard.

# Peer review: Referee response for version 2

**DOI:** 10.5256/f1000research.19170.r42131

**Published:** 2019-01-08

**Authors:** Stephanie Hicks

**Affiliations:** 1 Johns Hopkins Bloomberg School of Public Health (JHSPH), Baltimore, MD, USA

Thank you to the authors for their thoughtful responses. I appreciated the detail in version 2 of the manuscript.

I have read this submission. I believe that I have an appropriate level of expertise to confirm that it is of an acceptable scientific standard.

# Peer review: Referee response for version 1

**DOI:** 10.5256/f1000research.17256.r37228

**Published:** 2018-08-31

**Authors:** Stephanie Hicks

**Affiliations:** 1 Johns Hopkins Bloomberg School of Public Health (JHSPH), Baltimore, MD, USA

Freytag et al. have produced a nice research article on assessing methods for clustering scRNA-seq data from the 10x Genomics platform. I was excited to read the article to learn about what they recommend using. I have made some suggestions below for improvements that are mostly related to providing more intuition and higher-level summaries. This is mostly because as a user of these methods, at the end of the paper, I still felt a little confused about which method the authors would recommend using. I hope the authors can update the article with some of the suggestions:

 While the authors have provided detailed comparisons (running time, cluster stability, use of different aligners, different genes, etc), the biggest suggestion would be that the authors provide a higher-level summary of what the authors would suggest a user use to cluster his/her data. At the end of reading this paper, I felt a little overwhelmed at the amount of comparisons across various datasets. It's hard to look at Figs 1-7 and get an overall summary of which method to use. The authors do state in the abstract "We found that some methods, including Seurat and Cell Ranger, outperform other methods, although performance seems to be dependent on the complexity of the studied system", but it would be great if the authors could somehow provide a visual high-level summary of how they came to that conclusion, or elaborate in the discussion on that. 

 For the "gold standard" data, what was the percent of each human lung cell lines (HCC827, H1975, H2228) that were mixed together? Equal proportions? Was the reason you needed to use demuxlet was because the cell lines were mixed up for sequencing? It would be great if the authors could elaborate on the experimental design. 

 Is the "gold standard" data available with the SNVs called for each cell. It would be useful to have this count matrix and corresponding phenotypic information about each cell in a SingleCellExperiment object for others to have access to. 

 It would be great if the authors could include another example dataset with a batch effect in it or something with a slightly less clean design, given most datasets are not quite this "clean". Also, maybe different clustering methods would perform better / worse depending on they data contained rare vs common cell types or included more or less diversity. 

 There is a TENxPBMCsData package (https://github.com/kasperdanielhansen/TENxPBMCData) that has been submitted to Bioconductor (similar to the TENxBrainData). This includes all PBMC 10X datasets currently listed on their site and loads in a SingleCellExperiment object into R. For the Silver Standard Datasets, you might incorporate this into your workflow. 

 How did you (or Cell Ranger) deal with empty droplets or swapped barcodes on the 10x platform? This seems relevant for discovering cell types using some form of clustering.

 Supplemental Table 1 could use a caption and a label at the top saying "Supplemental Table 1". I had many tabs open with different supplemental figures and tables, and was getting confused about which was which one. 

 Why did Linnorm and Monocle "continually failed to run"? Did the authors contact the original authors of Linnorm and Monocle to determine if there was a problem with the actual software or if it was a problem with the implementation of the software? It would be great if the authors could elaborate. 

 I agree with this statement: " We concede that it is possible that more care in the upstream data handling and selection of parameters could result in different results." This is true for almost all benchmarking papers. Given the authors are working within the R/Bioconductor framework, it would be great if the authors could use something like SummarizedBenchmark (http://bioconductor.org/packages/release/bioc/vignettes/SummarizedBenchmark/inst/doc/SummarizedBenchmark.html) to keep track of these parameters. 

 Could the authors elaborate on how they decided which performance metrics to use? 

 What does this mean: "The impact of different aligners and preprocessing was assessed using all appropriate combinations of programs"? Could the authors be more specific?

 I'm a little concerned about how much the solutions differ between methods and parameter choices. I understand the point of this paper is to make comparisons between already published methods, but as the authors are now very familiar with these methods, it would be great if they could provide some more practical guidance. What would the authors suggest using? 

 Fig 1 -- Could the authors hypothesize on why Seurat, TSCAN, RCA, SC3, RaceID, RaceID2 are estimating so many clusters? Also, why does countClust tend to underestimate the number of clusters? It would be great if the authors could provide some intuition. 

 Fig 3 -- If I'm understanding, ascend and countClust produce clusters that are very different than the rest? 

 Thank you to the authors for making their code publicly available!

I have read this submission. I believe that I have an appropriate level of expertise to confirm that it is of an acceptable scientific standard, however I have significant reservations, as outlined above.

# Response

**Published:** 2018-12-11

**Authors:** Saskia Freytag

**Affiliations:** Walter and Eliza Hall Institute of Medical Research, Australia

*We would like to thank the reviewer for reviewing our manuscript and for their constructive comments. Below are point-by-point responses to the individual comments.*

 While the authors have provided detailed comparisons (running time, cluster stability, use of different aligners, different genes, etc), the biggest suggestion would be that the authors provide a higher-level summary of what the authors would suggest a user use to cluster his/her data. At the end of reading this paper, I felt a little overwhelmed at the amount of comparisons across various datasets. It's hard to look at Figs 1-7 and get an overall summary of which method to use. The authors do state in the abstract "We found that some methods, including Seurat and Cell Ranger, outperform other methods, although performance seems to be dependent on the complexity of the studied system", but it would be great if the authors could somehow provide a visual high-level summary of how they came to that conclusion, or elaborate in the discussion on that. 

*We have added a discussion section in which we summarize the results across all evaluations. This discussion section includes a visual high-level summary (Figure 9).*

 For the "gold standard" data, what was the percent of each human lung cell lines (HCC827, H1975, H2228) that were mixed together? Equal proportions? Was the reason you needed to use demuxlet was because the cell lines were mixed up for sequencing? It would be great if the authors could elaborate on the experimental design. 

*We mixed the cell lines in equal proportions. Due to using 10x Genomics technology, the cell lines were mixed up in the process but could be deconvoluted using demuxlet (ref?). We have elaborated on this further in the manuscript to clarify the experimental design.*

 Is the "gold standard" data available with the SNVs called for each cell. It would be useful to have this count matrix and corresponding phenotypic information about each cell in a SingleCellExperiment object for others to have access to. 

*We have made all datasets as SingleCellExperiment objects, including their phenotypic information, available on Github at https://github.com/bahlolab/cluster_benchmark_data . We have added information regarding the availability of all processed datasets to the manuscript.*

 It would be great if the authors could include another example dataset with a batch effect in it or something with a slightly less clean design, given most datasets are not quite this "clean". Also, maybe different clustering methods would perform better / worse depending on they data contained rare vs common cell types or included more or less diversity. 

*We agree that investigating the performance of clustering approaches on ”messy” scRNA-seq designs would be very interesting. However, this is beyond the scope of this paper, as it requires the application of sophisticated batch correction methods. Such methods should generally be performed by experts rather than beginners, who were the target audience of this paper. We have added a discussion to this effect.*

*Finally, in order to investigate whether methods perform better or worse in more or less diverse situations, one requires either simulations or mixture experiments. These were beyond the scope of this paper. However, we now refer the readers of our manuscript to the recent benchmarking study of scRNA-seq clustering methods by Duó et al, which investigates just such a scenario. For most methods they did not observe overt differences.*

 There is a TENxPBMCsData package (https://github.com/kasperdanielhansen/TENxPBMCData) that has been submitted to Bioconductor (similar to the TENxBrainData). This includes all PBMC 10X datasets currently listed on their site and loads in a SingleCellExperiment object into R. For the Silver Standard Datasets, you might incorporate this into your workflow. 

*We decided to incorporate all moderately large fresh PBMC samples included in the TENxPBMCs into our workflow. This also provided us with an opportunity to update the package versions for the individual clustering tools for our silver standard benchmarking and stability analyses.*

 How did you (or Cell Ranger) deal with empty droplets or swapped barcodes on the 10x platform? This seems relevant for discovering cell types using some form of clustering.

*We added the following explanation: “Cell Ranger filters any barcode that contains less than 10% of the 99th percentile of total UMI counts per barcode, as these are considered to be barcodes associated with empty droplets. The barcode by design can take one of 737,000 different sequences that comprise a whitelist. This feature allows the performance of error correction when the observed barcode does not match any barcode on the whitelist due to sequencing error.”*

 Supplemental Table 1 could use a caption and a label at the top saying "Supplemental Table 1". I had many tabs open with different supplemental figures and tables, and was getting confused about which was which one. 

*We added a caption on the top of all Supplemental Tables.*

 Why did Linnorm and Monocle "continually failed to run"? Did the authors contact the original authors of Linnorm and Monocle to determine if there was a problem with the actual software or if it was a problem with the implementation of the software? It would be great if the authors could elaborate. 

*Linnorm failed because its calculations would time out. Monocle failed because the dispersion could not be calculated. However, neither of the programs was tried using their newer package versions corresponding to R version 3.5.0 nor were any of the packages’ authors contacted. We have included a statement in the manuscript to this regard.*

 I agree with this statement: " We concede that it is possible that more care in the upstream data handling and selection of parameters could result in different results." This is true for almost all benchmarking papers. Given the authors are working within the R/Bioconductor framework, it would be great if the authors could use something like SummarizedBenchmark (http://bioconductor.org/packages/release/bioc/vignettes/SummarizedBenchmark/inst/doc/SummarizedBenchmark.html) to keep track of these parameters. 

*We did take a look at the SummarizedBenchmark package, but did not find it suitable for our needs. However, we understand the need to provide all parameters (including defaults) used in the individual analyses and thus have added additional files providing this information to the GitHub respository.*

 Could the authors elaborate on how they decided which performance metrics to use? 

*We used performance metrics commonly used in the clustering literature. We also made sure that the selected metrics were applicable in the absence of known cluster labels. Furthermore, they share the advantages of bounded ranges and no assumptions regarding cluster structures. Additionally they offer complementary insights. We have added this explanation to the manuscript.*

 What does this mean: "The impact of different aligners and preprocessing was assessed using all appropriate combinations of programs"? Could the authors be more specific?

*We meant to say that we assessed the impact of combinations of different aligners and preprocessing (i.e. CellRanger or scPipe) for all possible clustering methods. Some clustering methods, like ascend, failed to run for scPipe generated output and it was too challenging to run the CellRanger clustering approach on scPipe generated output.*

 I'm a little concerned about how much the solutions differ between methods and parameter choices. I understand the point of this paper is to make comparisons between already published methods, but as the authors are now very familiar with these methods, it would be great if they could provide some more practical guidance. What would the authors suggest using? 

*We suggest using several clustering methods ideally with multiple parameter choices in order to ensure that biological results are not artifacts of method or parameter choice. Unfortunately, we do not feel in a position to give specific practical advice for the specific use of individual methods, as optimal parameter choices depend on many different factors including the type of biological system studied.*

 Fig 1 -- Could the authors hypothesize on why Seurat, TSCAN, RCA, SC3, RaceID, RaceID2 are estimating so many clusters? Also, why does countClust tend to underestimate the number of clusters? It would be great if the authors could provide some intuition. 

*We believe that many methods tended to overestimate the number of clusters in the gold standard dataset, because the cell lines may be heterogeneous with regards to other biological factors, such as cell state. Consequently, in such a scenario methods may split cells of the same population but in different cell states into multiple clusters.*

*We have no intuition as to why countClust underestimates the number of clusters.*

 Fig 3 -- If I'm understanding, ascend and countClust produce clusters that are very different than the rest? 

*Yes that is correct.*

# Peer review: Referee response for version 1

**DOI:** 10.5256/f1000research.17256.r37231

**Published:** 2018-08-29

**Authors:** Shila Ghazanfar

**Affiliations:** 1 School of Mathematics and Statistics, University of Sydney, Sydney, NSW, Australia

Freytag and colleagues provide a comprehensive comparison of clustering methods - specifically designed for scRNA-Seq data - on data collected using the popular droplet-based 10x Genomics platform. A total of four datasets, comprising a Gold standard mixture of cell lines as well as three Silver standard PBMC datasets, were compared in terms of accuracy, stability as well as other metrics like runtime and ease of use. Freytag et al also perform an analysis to try to determine the factors influencing the resulting clusterings for the Silver standard datasets.

 It is a very challenging task to perform a comprehensive characterization and comparison of clustering methods on such types of high-dimensional data, due to the sheer number of choices that need to be made, the difficulty in establishing ideal performance, and the relative lack of ground truth. Freytag et al do a great job of addressing these challenges and working towards providing an overall recommendation of clustering methods for non-expert practitioners, while stressing the need for careful interpretation of such results. 

 With this in mind, I have some comments/suggestions, as well as a number of minor comments/suggestions, as follows:

 **Comments to authors**

 Linnorm and Monocle failed - expand on why? I understand that this is indeed a limitation especially for a non-expert practitioner, but it would be good to have an understanding towards what the issue might have been.

 Could use a flowchart to summarise the study and various comparisons, as well which methods could no longer be compared (e.g. methods that could not work within the scPipe framework).

 Different upstream data handling was performed for each clustering method. How much of a difference was observed just due to this preprocessing, as opposed to the actual clustering step? I understand that each method provides their own preprocessing as *part* of the method, but at least some of these methods would have been developed with plate-based and/or non-UMI-based scRNA-Seq in mind, so may not be intended for the context of 10x Genomics data. Again I understand that you're comparing methods 'out of the box' but it would be insightful to see what differences there are. I suggest a figure like an upsetR plot for the genes/cells filtered and a correlation heatmap of the expression values themselves.

 Could you summarise the distance metrics used in the clustering and if there is a general flavour to the clustering algorithm? e.g. hierarchical, k-means, density-based etc. How do these relate in terms of overall accuracy, stability and other metrics?

 Stability assessment - mentions that half of the 58,302 genes were randomly selected, but Table 1 says 24,654 total genes detected. There's a big discrepancy between these two so please clarify; if half of the 58,302 genes were selected then a large proportion of genes would have identically zero rows. Also Table 1 shows Dataset 3 had the highest number of 'total genes detected', so how was Dataset 1 the one with "most number of non-zero genes after filtering"?

 Run time section - What do you mean by 'overridden'? And for which aspects of the analysis steps was this done?

 Figure 4 - These boxplots show ARI among multiple clustering solutions, so a method that gives a consistently bad result is still high (e.g. in this case the RCA method). Suggest an analogous set of boxplots but with ARI_truth, is there a similar variability observed, as seen in these boxplots?

 Gene-wise stability analysis - I'm actually unsure how realistic this particular comparison is. It would be insightful to assess clusterings depending on different levels of gene filtering stringency (in the initial Cell Ranger read processing), or stringency on selection of features based on various criteria like highly variable genes.

 Figure 7 - Please clarify how 'total number of features' is a cell-specific quantity. Do you mean total number of non-zero features? Was this analysis also performed on the Gold Dataset and what overall similarities could be observed?

 Factors influencing clustering solutions - It would be interesting to consider the factors associated with 'correct' cluster assignment for cells. Optionally suggest to perform this for either the Gold Dataset or the Silver datasets and perform a logistic regression with the response being success/failure of a cell to belong to the cluster most associated with the 'true' cell type group. There is an added subtlety as far as matching clusters with cell type groups goes, but I think there are a few reasonable ways to perform this (e.g. assign candidate clusters to the 'true' groups by taking the higher proportion of cell overlap, and allow multiple candidate clusters to match to a single true group). Performing this kind of analysis could shed light on properties of cells that don't tend to cluster correctly, and if there is consistency in this across multiple disparate datasets.

 **Minor comments**

 Table 1 - countClust 'version' formatted with verbatim.

 Table 1 - I would suggest the 'properties' column could be better presented in a checklist format, with ticks/crosses for fulfilling various criteria listed.

 Section beginning "silver standard" - 10x is capitalised.

 Supplementary Figure 1 - legend fallen off panel a), needs a higher resolution or larger points

 NMI definition - trailing parenthesis in denominator

 typo - assess the effect**

 Figure 2a - I found this quite busy, hard to interpret. Suggest to add shading that covers the points for same method or to facet by dataset. I don't believe the ARI values are particularly comparable between datasets so I would prefer facetting by dataset.

 Figure 3 - rows/columns are ordered differently between panels, what's driving this difference?

 Supplementary Figure 3 was not mentioned in the main text

 Supplementary Figure 4 is a two page pdf, with the first page blank

 Figure 6 - Figure caption says Dataset 1 but reports 29,151 genes. Do you mean the Gold Dataset and 29,451 genes? If not, please clarify which data and how many genes.

 Discussion - One instance of "Seurat" is missing verbatim format

I have read this submission. I believe that I have an appropriate level of expertise to confirm that it is of an acceptable scientific standard, however I have significant reservations, as outlined above.

# Response

**Published:** 2018-12-11

**Authors:** Saskia Freytag

**Affiliations:** Walter and Eliza Hall Institute of Medical Research, Australia

*We would like to thank the reviewer for reviewing our manuscript and for their constructive comments. Below are point-by-point responses to the individual comments.*

 Linnorm and Monocle failed - expand on why? I understand that this is indeed a limitation especially for a non-expert practitioner, but it would be good to have an understanding towards what the issue might have been.

*Linnorm failed because its calculations would time out. Monocle failed because the dispersion could not be calculated. However, neither of the programs was tried using their newer package versions corresponding to R version 3.5.0. We have included a statement in the manuscript to this regard.*

 Could use a flowchart to summarize the study and various comparisons, as well which methods could no longer be compared (e.g. methods that could not work within the scPipe framework).

*While we were unable to summarize our study design effectively in a flowchart, we have summarized it in a table (see Supplementary Table 2). We hope that this will clarify the various assessments performed in this paper.*

 Different upstream data handling was performed for each clustering method. How much of a difference was observed just due to this preprocessing, as opposed to the actual clustering step? I understand that each method provides their own preprocessing as *part* of the method, but at least some of these methods would have been developed with plate-based and/or non-UMI-based scRNA-Seq in mind, so may not be intended for the context of 10x Genomics data. Again I understand that you're comparing methods 'out of the box' but it would be insightful to see what differences there are. I suggest a figure like an upsetR plot for the genes/cells filtered and a correlation heatmap of the expression values themselves.

*We agree that different data handling influences the performance of each clustering method, which were indeed designed with different single cell technologies in mind, and that the effect of this would be interesting to further investigate. However, the `black-box` nature of some of the investigated methods means that even recording these differences is challenging. Take Seurat as an example it is unclear whether to report the number of genes passing the filtering step or the number of genes that are used in the clustering.  Instead, we would like to refer you to the recent benchmarking study of clustering methods for scRNA-seq by Duó et al, where the authors investigated the effects of different gene filtering on clustering solutions.*

 Could you summarise the distance metrics used in the clustering and if there is a general flavour to the clustering algorithm? e.g. hierarchical, k-means, density-based etc. How do these relate in terms of overall accuracy, stability and other metrics?

*Thank you for the suggestion. We have updated the table summarizing the properties of the different clustering methods and added a discussion regarding how different flavors of clustering methods relate to overall performance (see Table 1 and Discussion).*

 Stability assessment - mentions that half of the 58,302 genes were randomly selected, but Table 1 says 24,654 total genes detected. There's a big discrepancy between these two so please clarify; if half of the 58,302 genes were selected then a large proportion of genes would have identically zero rows. Also Table 1 shows Dataset 3 had the highest number of 'total genes detected', so how was Dataset 1 the one with "most number of non-zero genes after filtering"?

*You are correct. We randomly selected half of 58,302 genes of which many were zero. We have since replaced this analysis, as per your suggestion, with an analysis that assesses stability when keeping only the top 10th, 20th, 30th, 40th, and 50th percentile of all genes including the ones not detected.*

*With regards to the number of detected genes in dataset 1 and dataset 3, indeed dataset 3 had more detected genes. Thank your for correcting this.*

 Run time section - What do you mean by 'overridden'? And for which aspects of the analysis steps was this done?

*We meant to say that a seed had been set to provide reproducibility of all parts of the analysis that involve randomness. This has been corrected in the manuscript.*

 Figure 4 - These boxplots show ARI among multiple clustering solutions, so a method that gives a consistently bad result is still high (e.g. in this case the RCA method). Suggest an analogous set of boxplots but with ARI_truth, is there a similar variability observed, as seen in these boxplots?

*Thank you for the suggestion, we have included a boxplot with ARI_truth.*

 Gene-wise stability analysis - I'm actually unsure how realistic this particular comparison is. It would be insightful to assess clusterings depending on different levels of gene filtering stringency (in the initial Cell Ranger read processing), or stringency on selection of features based on various criteria like highly variable genes.

*We have replaced the gene-wise stability analysis with an assessment of the performance when keeping only the top 10th, 20th, 30th, 40th, and 50th percentile of all genes (compare Figure 6). We think that this is more insightful as it is closer to filtering performed during analysis.*

 Figure 7 - Please clarify how 'total number of features' is a cell-specific quantity. Do you mean total number of non-zero features? Was this analysis also performed on the Gold Dataset and what overall similarities could be observed?

*Indeed we do mean the number of non-zero genes and we have replaced this in the figure with “number of detected genes”. We also include the same analysis on the gold standard dataset in the Supplementary (Supplementary Figure 11).*

 Factors influencing clustering solutions - It would be interesting to consider the factors associated with 'correct' cluster assignment for cells. Optionally suggest to perform this for either the Gold Dataset or the Silver datasets and perform a logistic regression with the response being success/failure of a cell to belong to the cluster most associated with the 'true' cell type group. There is an added subtlety as far as matching clusters with cell type groups goes, but I think there are a few reasonable ways to perform this (e.g. assign candidate clusters to the 'true' groups by taking the higher proportion of cell overlap, and allow multiple candidate clusters to match to a single true group). Performing this kind of analysis could shed light on properties of cells that don't tend to cluster correctly, and if there is consistency in this across multiple disparate datasets.

*We did perform the suggested analysis. However, results from this analysis did not give any insights beyond the already conducted analysis (see* https://github.com/SaskiaFreytag/cluster_benchmarking_code/tree/master/revision_figure *). Hence, we chose not to include this in the manuscript.*

 Table 1 - countClust 'version' formatted with verbatim.

*Thank you for noticing, this has been corrected.*

 Table 1 - I would suggest the 'properties' column could be better presented in a checklist format, with ticks/crosses for fulfilling various criteria listed.

*Table 1 is now Supplementary Table 1. Unfortunately, properties differ too much to adequately represent these in a checklist.*

 Section beginning "silver standard" - 10x is capitalised.

*Thank you for noticing, this has been corrected.*

 Supplementary Figure 1 - legend fallen off panel a), needs a higher resolution or larger points

*We have increased the resolution.*

 NMI definition - trailing parenthesis in denominator

*Thank you for noticing, this has been corrected.*

 typo - assess the effect**

*Thank you for noticing, this has been corrected.*

 Figure 2a - I found this quite busy, hard to interpret. Suggest to add shading that covers the points for same method or to facet by dataset. I don't believe the ARI values are particularly comparable between datasets so I would prefer facetting by dataset.

*We agree with the reviewer and now use faceting.*

 Figure 3 - rows/columns are ordered differently between panels, what's driving this difference?

*The difference by clustering on the similarity across methods, i.e. more similar methods are closer to each other. We have included a statement explaining this in the figure description.*

 Supplementary Figure 3 was not mentioned in the main text

*We now mention this Supplementary Figure.*

 Supplementary Figure 4 is a two page pdf, with the first page blank

*We have corrected this error.*

 Figure 6 - Figure caption says Dataset 1 but reports 29,151 genes. Do you mean the Gold Dataset and 29,451 genes? If not, please clarify which data and how many genes.

*Note that this figure has been replaced. We indeed meant Dataset 1, but with only half the genes.*

 Discussion - One instance of "Seurat" is missing verbatim format

*Thank you for noticing, this has been corrected.*

# Peer review: Referee response for version 1

**DOI:** 10.5256/f1000research.17256.r37232

**Published:** 2018-08-28

**Authors:** Joshua W. K. Ho

**Affiliations:** 1 Victor Chang Cardiac Research Institute (VCCRI), Darlinghurst, NSW, Australia

This paper presents a well-designed and comprehensive evaluation of widely used clustering algorithms for medium-sized 10x Genomics scRNA-seq data. Clustering is a highly active area of research in scRNA-seq data analysis. With so many published clustering tools available, it is often difficult to choose the most appropriate tool. This paper attempts to address this problem by systematically comparing the performance of 12 commonly used clustering tools. The evaluation results should serve as an important guide to bioinformatics practitioners. This paper is a very useful contribution to the field.

I have read this submission. I believe that I have an appropriate level of expertise to confirm that it is of an acceptable scientific standard.
